# Synergistic anticancer activity of antimicrobial peptide nisin and doxorubicin against breast cancer cells via modulation of membrane permeability

**DOI:** 10.1371/journal.pone.0352312

**Published:** 2026-06-23

**Authors:** Chanita Phetdee, Suwatjanee Naephrai, Malinee Pradain, Yanisa Panporm, Ubaid Ahmad, Panchika Prangkio

**Affiliations:** 1 Department of Chemistry, Faculty of Science, Chiang Mai University, Chiang Mai, Thailand; 2 Program in Biotechnology, Multidisciplinary and Interdisciplinary School, Chiang Mai University, Chiang Mai, Thailand; 3 Center of Excellence in Materials Science and Technology, Faculty of Science, Chiang Mai University, Chiang Mai, Thailand; University of Patras, GREECE

## Abstract

Breast cancer is the most frequently diagnosed cancer and remains the leading cause of death among females. Despite the rapid advancement in cancer therapy, the development of more effective therapeutic strategies and anticancer agents with a new mode of action remains a critical challenge. Combination of therapeutic agents is an attractive approach to enhance drug efficacy. Nisin, a cationic antimicrobial peptide, has been reported for its cytotoxicity against some cancer cell lines via several mechanisms, particularly membrane disruption. Nisin can bind preferentially to the negatively charged phospholipids, causing pore formation in cell membranes. In this study, we demonstrated that nisin exhibited membrane permeabilization preferentially with anionic lipids using liposomal leakage assay. Moreover, anticancer activity of nisin and doxorubicin (DOX) was investigated against two breast cancer cell lines, MCF-7 and MDA-MB-231, using MTT assay. Nisin demonstrated cytotoxic effect against breast cancer MCF-7 and MDA-MB-231, with IC_50_ values of 5–8 µM, while exhibiting lower cytotoxicity toward normal cells. Based on Bliss independence analysis, the synergistic effect between nisin and DOX was markedly observed in MCF-7 when treated with 10 μM nisin and 1 μM DOX for 24–48 h treatment. Furthermore, as demonstrated by fluorescent-based high-content analysis, nisin clearly caused cell membrane permeability and promoted DOX-induced DNA damage in both cell lines. Flow cytometry with Annexin-V staining revealed that co-treatment of nisin and DOX significantly enhanced apoptosis, as compared to individual treatments, particularly in MCF-7 cells, suggesting a key mechanism of action for anticancer activity. Cationic nisin can interact with biological membrane and modulate membrane permeability and fluidity, consequently facilitating DOX entry, inducing apoptosis and DNA damage. Overall, this study demonstrates that the combination of nisin and DOX could offer a new therapeutic approach for breast cancer treatment with a reduced chemotherapeutic drug dosage.

## Introduction

Breast cancer is one of the most prevalent cancers among females, exhibiting a significant incidence and mortality rate worldwide [1,2]. Over several decades, the overall breast cancer death rate has continuously declined by 44% due to improved screening and multimodal treatments, including surgery, radiation, and chemotherapy [3,4]. However, breast cancer remains a major threat to women’s health and is the leading cause of female cancer mortality. Cancer cells exhibit abnormal behaviors, producing growth signals, escaping growth-inhibitory signals and evading programmed cell death, which lead to uncontrolled proliferation, invasion and metastasis [5–7].

Chemotherapy, utilizing cytotoxic agents such as doxorubicin (DOX), actinomycin D, and docetaxel, is a fundamental approach for various types of cancer [8–10]. However, conventional chemotherapeutics commonly show non-specific toxicity, affecting healthy cells and resulting in severe side effects [11,12]. Moreover, cancer cells commonly develop multidrug resistance (MDR) through the enhanced activity of drug-efflux pumps, resulting in a decrease in the effective intracellular drug concentration [13,14]. These issues underline the critical need to find alternative treatment candidates or adjunctive treatments that can specifically target cancer or enhance the efficacy of conventional drugs while allowing the use of lower dosages [15].

Due to the molecular complexity of cancer, combination of anticancer drugs becomes necessary to improve long-term prognosis and reduce progression of the disease [16,17]. This approach involves administration of multiple drugs concurrently or sequentially by targeting several components of different cancer pathways, which can help overcome drug resistance and reduce systemic toxicity [18]. An attractive option is to use antimicrobial peptides (AMPs), naturally occurring small proteins or peptides that are essential for the innate immune defense systems of several organisms against bacteria, fungi, and viruses [19]. In addition, AMPs have been investigated as anticancer agents due to their positive charges and amphiphilic nature. They can selectively bind to negatively charged cancer cell membranes via electrostatic interaction, and some AMPs are involved with various cellular processes within cancer cells, such as inducing apoptosis and inhibiting DNA synthesis by binding to specific enzymes [20]. Thus, the use of AMPs individually or in combination with other compounds has gained interest as an alternative to conventional chemotherapy [21,22].

Nisin, a polycyclic peptide composed of 34 amino acids, is one of the AMPs produced by *Lactococcus lactis,* commonly used as a food preservative in dairy products. Recently, nisin has been reported for its cytotoxic effect on some cancer cells [23,24] and antimicrobial activity against a wide range of Gram-positive bacteria [25,26]. The mode of action of nisin is based on the electrostatic interactions with the negatively charged bacterial membrane through Lipid II, which is a precursor for bacterial cell wall synthesis, causing cell membrane permeability and membrane disruption [27]. Thus, the pore-forming characteristic of nisin establishes it as a promising membrane-active agent to exert biological activity at the cell membrane. In addition to antimicrobial activity, nisin has been reported to exhibit anticancer and immunomodulatory properties in various cancer cell lines, such as head and neck squamous cell carcinoma (HNSCC), breast cancer cells (MCF-7), liver cancer cells (HepG2) and colon cancer cells (HT-29 and HCT-116) [28–31].

Although, the anticancer potential of nisin has been previously reported, the precise biophysical mechanisms by which nisin modulates membrane permeability to enhance the efficacy of conventional chemotherapeutics remain poorly understood [32]. Most studies have focused on the individual cytotoxicity of nisin, while its potential as a membrane-active adjuvant to overcome the uptake barriers of DOX has not been comprehensively validated across different molecular subtypes of breast cancer. Here, the nisin-DOX combination is selected based on their complementary modes of action. We hypothesize that nisin can interact with the negatively charged surface of targeted cancer cells via electrostatic interaction, inducing pore formation and enhancing anticancer drug uptake, ultimately resulting in cell death at reduced dosages.

Beyond electrostatic attraction to negatively charged surface molecules, cancer cell selectivity is influenced by multiple biological factors including membrane fluidity, cholesterol content, expression of receptors, metabolic state, and the heterogeneous nature of the tumor microenvironment [33]. Tumor heterogeneity leads to variations in membrane composition across different breast cancer subtypes, such as the hormone-responsive MCF-7 and the triple-negative MDA-MB-231. These characteristics can strongly affect therapeutic response by modulating cellular uptake, membrane permeability and susceptibility to cell death pathways. For instance, the differences in membrane dynamics and lipid packing may influence the peptide-induced pore formation, consequently affecting the enhanced cytotoxic effect of drug [34].

This study elucidates the mechanisms underlying a previously unexplained observation by providing comprehensive mechanistic evidence for enhanced therapeutic efficacy when used in combination. The preferential membrane permeabilization activity of nisin toward anionic lipids was studied using a dye leakage assay in liposomes, providing the biophysical basis for its cancer cell selectivity. We then evaluated the combinatory effect of nisin and DOX against two functionally distinct breast cancer cell lines, the hormone-responsive MCF-7 and the triple-negative MDA-MB-231. Moreover, Bliss independence analysis was utilized to quantitatively confirm the synergy and validate the hypothesized mechanism by demonstrating that the nisin in combination with DOX significantly enhances cellular uptake of DOX, promotes DOX-induced DNA damage, and dramatically increases apoptosis at concentrations below the toxic dosage for normal cells. Our findings define a clear therapeutic strategy where nisin acts as a membrane-permeabilizing adjuvant to improve the therapeutic index of DOX, particularly in hormone receptor-positive breast cancer.

## Materials and methods

### Materials

Nisin A from *Lactococcus lactis,* DOX, 5(6)-Carboxyfluorescein (CF) and 4-(2-hydroxyethyl) piperazine-1-ethanesulfonic acid (HEPES) were purchased from Sigma-Aldrich (St. Louis, MO, USA). 1-palmitoyl-2-oleoyl-glycero-3-phosphocholine (POPC), 1-palmitoyl-2-oleoyl-sn-glycero-3-phosphoethanolamine (POPE), 1-palmitoyl-2-oleoyl-sn-glycero-3-phospho-L-serine (POPS) and 1-palmitoyl-2-oleoyl-sn-glycero-3-phospho-rac-glycerol (POPG) were purchased from Avanti Polar Lipids (Alabaster, AL, USA). Lipid stock solutions were prepared in chloroform and stored at −20 ºC until used. Phosphate-buffered saline (PBS) was purchased from Gibco-Invitrogen (Carlsbad, CA, USA). 3-(4, 5-dimethyl-2-thiazolyl)-2, 5-diphenyl tetrazolium bromide (MTT) was obtained from PanReac Applichem (Darmstadt, Germany).

### Preparation of nisin

Nisin was dissolved in 0.02 M HCl to obtain a concentration of 40 mg/mL and stirred continuously for 1 min. The suspension was centrifuged at 1000 × g for 10 min. Then, the pellets were discarded, and the supernatant was stored at −20 ºC until use. Nisin concentration was determined by Bradford assay [35]. Bovine serum albumin (BSA) standard or nisin was added into each well of 96-well plate containing 200 µL of Coomassie Reagent. The plate was incubated for 15 min at room temperature. The absorbance was measured at 595 nm by microplate reader (SpectraMax i3X, Molecular Devices, San Jose, CA, USA).

### Cell culture

Human breast cancer cell lines (MCF-7 and MDA-MB-231) and the mouse fibroblast L929 were obtained from ATCC (Manassas, VA, USA). The non-cancerous human keratinocyte (HaCaT: iCell-h066) cells were obtained from iCell Bioscience Inc. (Shanghai, China). Cells were cultured in Dulbecco’s modified eagle medium (DMEM, Gibco, Grand Island, NY, USA) supplemented with 10% FBS (Sigma-Aldrich), 100 U/mL penicillin (Gibco) and 100 μg/mL streptomycin (Gibco) under humidified conditions with 5% CO_2_ supply at 37 °C. All cell lines were routinely monitored throughout the study to confirm that they remained free of mycoplasma contamination.

### Liposome preparation and physicochemical characterization

Liposomes were prepared using the thin-film hydration method [36]. Lipid mixture of POPC and POPE (or POPC/POPG, POPC/POPS) at the 1:1 mol ratio was dissolved in chloroform and the solvent was removed under vacuum by a rotary evaporator, resulting in a thin and homogeneous lipid film. The lipid film was hydrated with 20 mM HEPES, pH 7.4 buffer containing 80 mM fluorescent CF dye. The lipid suspension was freeze-thawed 15 times, followed by extrusion 21 times using an Avanti mini-extruder (Alabaster, AL, USA) through a polycarbonate membrane (0.1 µm pore size) [37,38]. The unencapsulated dye was removed through size exclusion chromatography, Sephadex G-25 in PD-10 column (GE Healthcare, Buckinghamshire, UK), which was initially equilibrated with 20 mM HEPES, pH 7.4. The liposomes were diluted 20 times with deionized water before size and zeta potential analyses. The mean particle size and zeta potential were determined by dynamic light scattering using a Zetasizer Nano ZS (Malvern Instruments Ltd., Malvern, UK).

### Determination of membrane permeabilization

Nisin-induced membrane permeabilization was assessed by leakage of CF dye from liposomes with different lipid compositions, following protocols from previous studies [39]. The concentrations of phospholipids were estimated by phosphorus determination through acidic digestion [40,41]. The liposomes were diluted with HEPES buffer at pH 7.4 before membrane permeabilization determination. The lipid concentration at 60 µM and nisin at 10 µM were used for the dye leakage assay. The quenching concentration of CF used for the assay was 80 mM. To determine pore-forming ability, dye leakage was monitored by measuring the fluorescence intensity of all liposomes with excitation and emission wavelengths of 492 and 517 nm, respectively, using a microplate reader (SpectraMax i3X, Molecular Devices, San Jose, CA, USA). The reading was stabilized for 30 min after each addition of nisin. Triton X-100, 1% was added at the end of each experiment to disrupt the vesicles, and the resulting fluorescence was taken as 100% leakage value. For kinetic analyses, the percentage of dye leakage was determined by the equation (1):


$$\begin{array}{c} {\text{Leakage }(\%)={(\text{F}}_{\text{t}}-{\text{F}}_{\text{0}})\times \text{100}/({\text{F}}_{\text{m}}-\text{F}}_{\text{0}}), \end{array}$$ (1)

where F_0_ is the initial fluorescence, F_m_ is the total fluorescence observed upon addition of 1% Triton X-100, and F_t_ is the fluorescence observed after adding nisin at each time point.

### MTT assay

Cell viability was evaluated using the colorimetric method of MTT assay in MDA-MB-231, MCF-7; human breast cancer cell lines compared with fibroblast L929 cell line and HaCaT human cell line. The cells were transferred to a 96-well plate at a density of 2 x 10^4^ cells/well and allowed to attach for 24 h. Cells were treated with 100 µL of final concentrations of 0.1–20 µM of nisin alone, DOX alone and nisin-DOX combination diluted in serum-free DMEM medium and maintained for 24 h at 37 ºC in a humidified incubator with 5% CO_2_ atmosphere. To perform the assay, 100 µL serum-free culture medium containing 1 mM of MTT was added to each well after removal of cell supernatant. The plates were incubated for 3 h at 37 ºC, and the dark blue formazan crystals were dissolved by adding 100 µL DMSO to each well. After a 15 min incubation in the dark, absorbance was measured at 540 nm using a microplate reader. Cells incubated with fresh serum-free DMEM were used as a negative control. The percentage of cell viability was determined according to the equation (2):


$$\begin{array}{c} \text{Cell v}\text{iability }(\%)=\left[\frac{{\text{A}}_{\text{540}}(\text{drugs})}{{\text{A}}_{\text{540}}(\text{negative control})}\right]\times \text{100}, \end{array}$$ (2)

The nonlinear curve-fitting program (GraphPad Software Inc., San Diego, CA, USA) was used to determine an IC_50_ value. The data of viability of cells treated with nisin were fitted to log (inhibitor) vs. normalized response model that can be calculated by the equation (3) to obtain IC_50_ values.


$$\begin{array}{c} \text{Y}=\text{100}/(\text{1}+\hat{\text{10}}\;(\text{X}-\text{LogIC50})), \end{array}$$ (3)

### Statistical analysis

The statistical analysis was performed by one-way analysis of variance (ANOVA) followed by Tukey’s post-hoc test for multiple comparisons using GraphPad Prism (Version 10.1.1) to determine significant differences between all experimental groups, when p < 0.05 was accepted as the lowest level of statistical significance. To study the synergistic effect of nisin and DOX, MCF-7 and MDA-MB-231 cell lines were treated with nisin in combination with DOX. Bliss independence drug interaction analysis was used to analyze drug combination data of the independent events [42]. At designated doses of the component drugs, the combined inhibition rate ${\hat{y}}_{ab}$ can be predicted by the equation (4):


$$\begin{array}{c} {\hat{y}}_{ab}=y_{a}+y_{b}-y_{a}y_{b},\text{0}\leq y_{i}\leq ,i=a,b\;or\;ab, \end{array}$$ (4)

where $y_{a}$ and $y_{b}$ are the observed inhibition rates with drug A alone at dose a and drug B alone at dose b. The difference of the experimentally observed activity of drug combination $y_{ab}$ and predicted activity of drug combination ${\hat{y}}_{ab}$ is used to indicate drug combination effects following these criteria (5):


$$\begin{array}{c} y_{ab}-{\hat{y}}_{ab}\left\{ \begin{array}{c} \;\;\;>\text{0}\;\text{synergism }\\ \;\;\;=\text{0}\;\text{independent}\\ \;\;\;<\text{0}\;\text{Antagonism} \end{array}\right.\; \end{array}$$ (5)

### Cellular uptake of DOX

MCF-7 cells were seeded in the 96-well plate at a density of 2 x 10^4^ cells/well and allowed to attach for 24 h. The cells were incubated with 10 and 20 µM of nisin and 1 µM of DOX and nisin in combination with DOX diluted in DMEM and incubated at 37°C for 24 h. Cells incubated with medium were used as a negative control. The medium was removed and discarded, and the cells were washed with PBS and fixed with 4% (w/v) paraformaldehyde solution for 15 min at room temperature. Nuclear staining was performed by Hoechst 33342 for 20 min. The fluorescence intensity of cells after treatment was measured with EX/EM wavelengths of 520 and 600 nm, respectively. Then, the fluorescent images of cells were analyzed using confocal microscope (Leica Stellaris 5, Leica Microsystems, Wetzlar, Germany).

### DNA damage analysis

DNA damage assay was performed according to the manufacturer’s instruction of the HCS DNA Damage Kit (Invitrogen, Carlsbad, CA, USA). MCF-7 and MDA-MB-231 cells were seeded in the 96-well plate at a density of 2 x 10^4^ cells/well for 24 h. The medium was replaced with 100 µL of 10 µM of nisin, 1 µM of DOX and nisin in combination with DOX diluted in DMEM. After 24 h of incubation, 50 µL of Image-IT® Dead Green™ viability stain solution was added and incubated for 30 min under normal cell culture conditions. Then, the cells were fixed in a 4% paraformaldehyde solution for 15 min at room temperature. The cells were washed with PBS once before being permeabilized with Triton X-100 solution for 15 min at room temperature. The cells were rinsed with PBS once before adding 1% BSA blocking solution. The blocking solution was removed after 1 h at room temperature, and 50 µL of phosphorylated H2AX (pH2AX) antibody solution (primary antibody) was added to each well and incubated for 1 h at room temperature. After rinsing with PBS, 50 µL of the cells were incubated with secondary antibody solution (eFluor 660 anti-mouse IgG and Hoechst 33342) for 1 h at room temperature. After incubation, the cells were washed three times with PBS and imaged using high-content screening microscope, imageXpress Micro4, Molecular Devices, San Jose, CA, USA). DNA double-strand breaks and cell membrane permeability were measured based on fluorescence detection.

### Apoptosis assay

An apoptosis assay was performed according to annexin V-FITC apoptosis detection kit (Dojindo, Kumamoto, Japan). Briefly, MCF-7 and MDA-MB-231 cells were seeded to a 6-well plate at a density of 3 × 10^5^ cells/well and allowed to attach for 24 h. The medium was replaced with 10 or 20 µM nisin and 1 µM DOX and nisin in combination with DOX diluted in DMEM medium and incubated for 24 h at 37 ºC in a humidified incubator with 5% CO_2_ atmosphere. The cells were harvested, washed in PBS and resuspended in 100 µL of binding buffer (0.01 M HEPES, pH 7.4, 0.14 M NaCl, and 2.5 mM CaCl_2_ solution) and incubated with 5 µL of Annexin V-FITC and PI staining solution for 15 min in the dark. Then, the cell suspension was mixed with 400 µL of binding buffer on ice and analyzed with a flow cytometer (CyAn ADP Analyzer, Beckman Coulter, Brea, CA, USA). Cells incubated with medium were used as a negative control.

## Results and discussion

### Nisin is likely to induce dye leakage from negatively charged membrane

Nisin is an antimicrobial peptide known to exert its biological activity by pore formation and membrane disruption. Membrane permeabilization of peptides depends on various factors such as lipid compositions, size and liposome surface charge. The surface charge of lipids is a key factor governing peptide-membrane interaction [43,44]. In this study, liposomes prepared from three different phospholipid compositions, containing zwitterionic POPC and lipids with different head groups at 1:1 molar ratio, including POPE, POPG and POPS were used for dye leakage assay to compare the effect of charges on membrane permeabilization by nisin. The zwitterionic POPC and POPE are major phospholipid component of cell membranes, commonly used to mimic biological membrane, while POPG and POPS represent amphiphilic phospholipids, which are negatively charged at neutral pH (see Supplementary Material, S1 Fig). Table 1 shows the average size of the liposomes of 130−150 nm which was acceptable for use as a lipid model for leakage assay [45]. The zeta potential of POPC:POPE liposomes was roughly −30 mV and became more negative with increasing content of negatively charged lipids (POPG or POPS) in the liposome compositions.

**Table 1:** Mean diameter and zeta potential of liposomes with different lipid compositions and the maximum of dye leakage (%) upon addition of nisin at 10 µM ^a.^

Lipid composition (molar ratio)	Mean diameter (nm)	Zeta potential (mV)	Max. dye leakage (%) (from 600–2400 s period)
POPC:POPE (1:1)	131.53 ± 0.13	−29.90 ± 0.17	−79.11% ± 10.11
POPC:POPG (1:1)	127.96 ± 2.45	−50.70 ± 0.80	46.40% ± 6.18
POPC:POPS (1:1)	149.63 ± 1.11	−65.40 ± 0.43	56.28% ± 4.75

^a^Values represent the mean ± SEM (n = 3).

**Fig 1:**
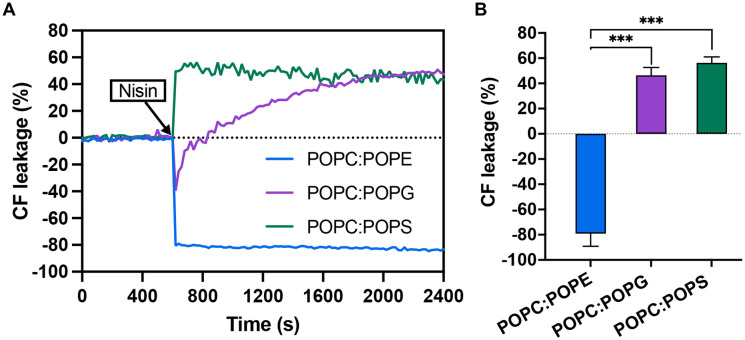
Nisin-induced dye leakage of CF from liposomes. **(A)** CF leakage (%) from liposomes with different compositions. The fluorescence intensity was continuously measured every 20 s for nisin with the liposomes. Triton X-100 (1%) was used as a positive control (100% CF leakage). **(B)** Maximal CF leakage (%) in different lipid membranes containing POPC:POPE, POPC:POPG and POPC:POPS at the 1:1 mol ratio upon the addition of nisin at 10 µM. The % leakage of each condition was averaged from 600–2400 s period when the leakage reached plateau. Values represent the mean ± SEM (n = 3).

Membrane permeability was assessed using a liposome leakage assay with self-quenched CF dye encapsulated at 80 mM. At this concentration, CF fluorescence is quenched inside the intact liposomes. Membrane disruption caused CF dye release into the external buffer, resulting in an increase in fluorescence intensity [46]. The specific concentrations utilized in the liposome leakage assay—60 µM lipid and 10 µM nisin were selected based on optimized biophysical parameters established in the membrane permeabilization study. A phospholipid concentration of 60 µM provides an optimal range for signal detection of fluorescent dye leakage. The nisin concentration of 10 µM is in the same range as that used in the cellular study. Upon addition of Triton X-100, 1%, the liposomal membrane was completely lysed, causing 100% dye leakage. For the liposomes composed of zwitterionic POPC and POPE, nisin did not cause dye leakage, but the reduction of fluorescence was rather observed upon addition of peptide, possibly due to aggregation of liposomes and interference of nisin with CF dye. On the other hand, the liposomes containing negatively charged lipids demonstrated a gradual increase in % leakage upon the addition of nisin, especially POPC:POPG, as illustrated in Fig 1. This is likely due to the electrostatic interactions between the negatively charged lipids and the positively charged nisin. These findings are consistent with previous studies showing nisin binding to giant unilamellar vesicle (GUV) containing negatively charged phospholipids [47]. The membrane permeabilization was slightly greater in liposomes containing POPC:POPS with dye release up to 56%. This could be attributed to more negative charges of POPC:POPS when compared to POPC:POPG liposomes. In addition, the selective binding and permeabilization effect of AMPs on cellular membranes is accounted for other physical parameters, such as the density of surface charges, membrane curvature, and lipid arrangement, all of which are modulated by the presence of charged lipids [48]. Overall, our findings suggest that the electrostatic interactions between nisin and negatively charged membranes along with other membrane characteristics contribute to nisin-induced membrane disruption. Based on this finding, we hypothesized that cationic nisin may exert cytotoxic effect in cancer cells that possess a higher density of negative charges than normal cells.

### Nisin exhibited cytotoxic effect on breast cancer cells

To examine cytotoxicity against breast cancer, the two commonly used human breast cancer cell lines with distinct characteristics, MCF-7 and MDA-MB-231 cell lines were treated with nisin for 24 h, 48 h and 72 h, in comparison with the normal HaCaT keratinocytes. Cell viability was quantified by MTT assay. MCF-7 and MDA-MB-231 represent different breast cancer subtypes with distinct therapeutic responses. MCF-7 represents luminal A breast cancer subtype which is a hormone-responsive cancer, expressing estrogen and progesterone receptors (ER^+^ and PR^+^). In contrast, MDA-MB-231 belongs to a triple-negative breast cancer cell line, lacking estrogen, progesterone and HER2 receptors, which is more aggressive and therapy-resistant cancer [49].

Here, we demonstrate cytotoxic effect of nisin against MCF-7 and MDA-MB-231 cell lines after 24-h exposure with the IC_50_ values of approximately 7.78 µM and 4.66 µM, respectively, which were lower than that of non-cancerous fibroblast L929 and HaCaT cell lines. Nisin exhibited greater cytotoxic effects toward cancer cells than normal cells, suggesting the potential selectivity of nisin against malignant cells. The previous studies exhibited that the cytotoxic effect of nisin on MCF-7 cells was shown predominantly at high doses of the peptide, with an IC_50_ value of 105.46 µM [30], which is significantly greater than the IC_50_ observed in this work. On the other hand, DOX was cytotoxic to breast cancer cells but also showed toxicity toward both normal cell lines, especially with human keratinocyte cell line, indicating a lack of cell-type selectivity. The IC_50_ value of free DOX in the HaCaT cell line was 1.40–2.90 µM after 24–72 h of incubation. This result is consistent with previous report showing an IC_50_ of 2.61 µM under the same exposure time in comparison with cancer cells [50]. All IC_50_ values of nisin and DOX for breast cancer and normal cell lines were summarized in Table 2. Dose-response curves of different cell lines were shown in Supplementary Material, S2 Fig.

**Table 2:** Mean IC_50_ values of nisin and DOX for different cell lines ^a.^

Samples	Cell lines	IC_50_ (μM)			Selectivity index (SI)		
		24 h	48 h	72 h	24 h	48 h	72 h
Nisin	MCF-7	7.78 ± 1.99	4.44 ± 1.13	6.81 ± 2.10	>2.57	>4.50	>2.94
	MDA-MB231	4.66 ± 0.79	3.41 ± 0.64	3.27 ± 0.46	>4.29	>5.86	>6.12
	Fibroblast	19.27 ± 6.16	20.02 ± 3.63	20.49 ± 2.19	–	–	–
	HaCaT	>20	>20	>20	–	–	–
DOX	MCF-7	27.87 ± 2.27	14.42 ± 2.16	8.79 ± 1.24	0.10	0.13	0.16
	MDA-MB231	7.06 ± 2.42	5.45 ± 2.03	3.70 ± 1.44	0.41	0.34	0.46
	Fibroblast	>20	10.10 ± 3.20	10.23 ± 5.62	–	–	–
	HaCaT	2.90 ± 0.57	1.84 ± 0.74	1.40 ± 0.42	–	–	–

^a^Values represent the mean ± SEM (n = 5).

As a pore-forming peptide, nisin has both cationic and hydrophobic characteristics that can interact with negatively charged headgroups and the lipophilic tail of phospholipids in cell membrane phospholipids [51]. Electrostatic interactions may play a major role in the selective cytotoxicity of this peptide. To evaluate therapeutic potential of the drug based on the IC_50_ values, we determined the selectivity index (SI) for nisin against both cancer cell lines by calculating the ratio between the IC_50_ values in a non-tumorigenic human cell line (HaCaT) to that in each cancer cell line under the same treatment conditions. SI values greater than 2 are typically considered high selectivity for the cancer cells, while a value lower than 2 indicates the general toxicity [52,53]. We found that nisin exhibited SI for MCF-7 and MDA-MB-231 cells in the range of 2.57–4.50 and 4.29–6.12, respectively, implying moderate selectivity of nisin for both cell lines. In contrast, the SI of DOX is generally low (SI < 2), indicating poor selectivity between cancer cells and normal cells, resulting in potential off-target toxicity. Previous studies identified that nisin exhibited minimal cytotoxicity against MCF-7 and MDA-MB-231 cell lines primarily by inducing apoptosis, inhibiting reactive oxygen species (ROS) production and modulating cell cycle processes [54].

Based on our findings, MDA-MB-231 cells were more sensitive to nisin treatment than MCF-7 cells, which typically exhibit a slower growth rate [55]. However, MCF-7 cells became more responsive after 48-h and 72-h treatment. Despite its moderate efficacy, the IC_50_ values of nisin against both breast cancer cell lines were lower than that of normal cells. This may be attributed to the difference in cell membranes of normal cells and cancer cells, which contain more negative surface charges on the membrane due to higher expression of negatively charged molecules [56,57]. Moreover, previous studies revealed that MDA-MB-231 cells exhibit an active signaling pathway, which facilitates increased secretion of inflammatory cytokines and provides resistance to TNF-induced apoptosis. This pathway is less functional in MCF-7 cells due to a lower in transcription factor protein complexes that regulate DNA transcription, cytokine synthesis, and cell survival [58]. Thus, the therapeutic effect of nisin in MDA-MB-231 was slightly greater than that of MCF-7. Nisin is likely to interact with cancer cell membranes, indicating a potential selectivity of nisin against breast cancer cells over normal cells.

### Nisin enhanced anticancer activity of DOX in MCF-7 cells

To enhance the therapeutic potential and overcome drug resistance of the anticancer drugs, the combination of nisin with chemotherapeutic drug, DOX, was used to treat both breast cancer cell lines: MCF-7 and MDA-MB 231, using nisin at 1–10 μM and DOX at 0.1–20 μM for 24, 48 and 72 h. We investigated drug interactions, particularly to determine whether nisin and DOX act synergistically, independently, or antagonistically, using Bliss independence analysis [42].

**Fig 2:**
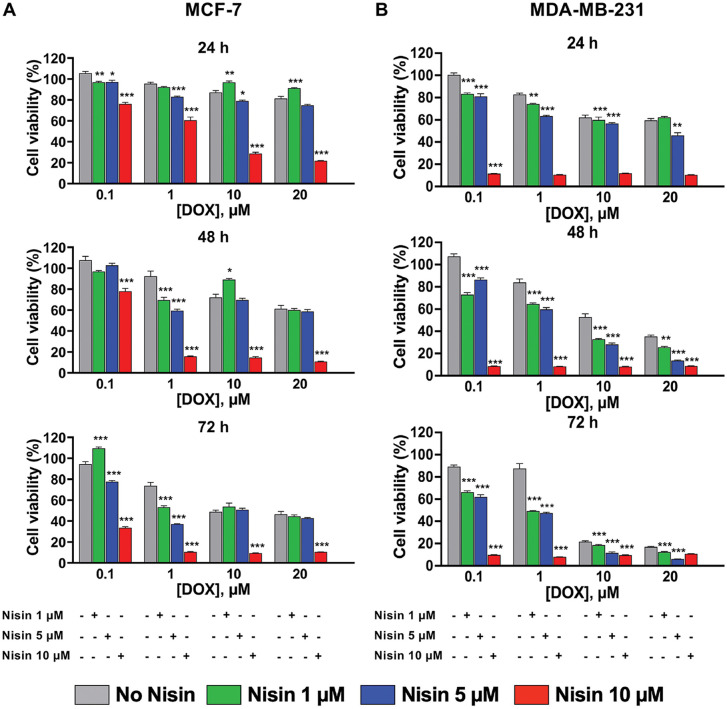
Cell viability (%) of breast cancer cell lines after treatment with combination of nisin and DOX. **(A)** MCF-7 and **(B)** MDA-MB-231 after treatment with combination of nisin and DOX at various concentrations and periods for 24 h, 48 and 72 h. Cell viability was determined by MTT assay. ^*^P < 0.05; ^**^P < 0.01; ^***^P < 0.001 compared with DOX alone. Values represent the mean ± SEM (n = 5).

**Fig 3:**
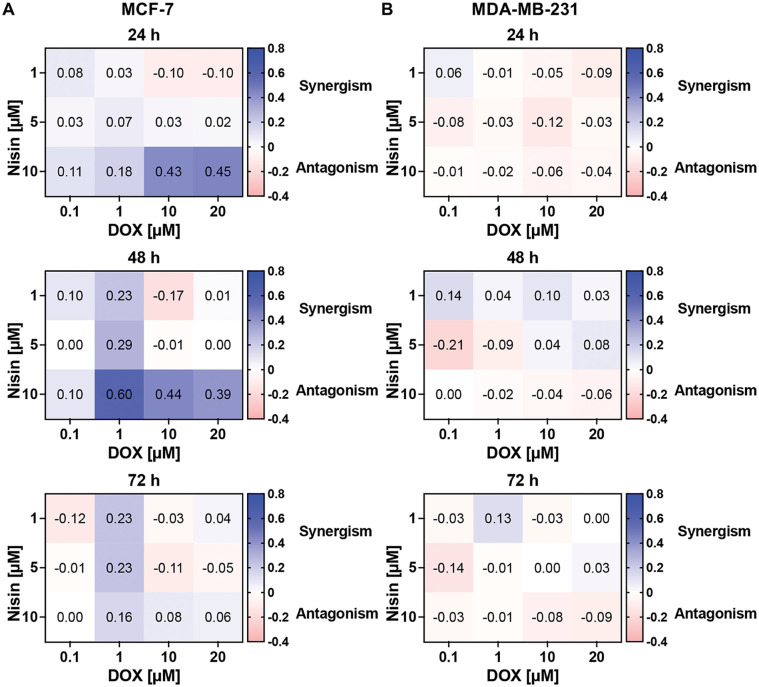
Combination effect of nisin and DOX treated with two breast cancer cell lines. Combination effect in **(A)** MCF-7 and **(B)** MDA-MB-231 at various concentrations and periods for 24 h, 48 h and 72 h. Combination effect was analyzed from the calculated %cell death from independent experiments (n = 5) using Bliss independence drug interaction analysis, when ΔY_ab_ > 0, = 0, and <0 represent synergistic, independent, and antagonistic effects, respectively.

As a result, an individual treatment of nisin or DOX exhibited dose-and time-dependent cytotoxicity against both MCF-7 and MDA-MB-231 cell lines. For co-treatment of nisin and DOX, the %cell viability of MCF-7 cells decreased significantly as compared with individual treatment of nisin or DOX, especially at high concentrations of nisin (10 μM) and DOX (10 μM), as shown in Fig 2A. The anticancer effect against MCF-7 cells was remarkably enhanced when treated with DOX (1−20 μM) in the presence of nisin at 10 μM, especially at 24−48 h. Based on the Bliss independence analysis, the combined effect of nisin and DOX in MCF-7 was synergistic rather than independent or antagonistic as indicated by Bliss interaction index (ΔYab > 0) shown in Fig 3A.

In the case of MDA-MB-231, a combination of nisin and DOX induced cytotoxicity, but the synergistic cytotoxic effect between two drugs was overall less pronounced as compared to MCF-7 (see Fig 2B). The anticancer effect of nisin and DOX was rather independent of each other, especially at 72 h exposure (ΔYab ~ 0), whereas a slight antagonistic effect was observed at 24 and 48 h as shown in Fig 3B. Previous studies have reported that MDA-MB-231 cells exhibit elevated expression of efflux pumps which might lead to decreased intracellular drug accumulation and reduced the synergistic effects [59]. Additionally, another study suggested that MDA-MB-231 cell membranes contain higher cholesterol and lipid rafts compared to MCF-7 cells, which may affect membrane integrity and permeability induced by nisin [60].

Nisin is likely to bind with negatively charged membrane of cancerous cells, causing pore-formation and phospholipid rearrangement, thus increasing uptake of DOX by cancer cells. Although MCF-7 cells are generally considered more sensitive to DOX due to lower drug efflux and resistance mechanisms, in this case, DOX exhibited less cytotoxicity against MCF-7 than MDA-MB-231, suggesting additional factors such as differential signaling pathways or metabolic adaptation. However, other studies have reported that MDA-MB-231 cells are more sensitive to DOX, which is associated with differential modulation of epithelial-mesenchymal transition [61]. The variations in drug response may also depend on other experimental factors such as cell cycle, culture conditions, number of cells and evaluation methods [62]. Nevertheless, nisin clearly exhibited cytotoxic effects against both cell lines and could be potentially used as an anticancer agent in combination with other drugs.

Based on the cytotoxicity studies, co-treatment of nisin and DOX exerted cytotoxic effects against both breast cancer cell lines. Our findings showed that a combination of nisin and DOX exhibits a markedly synergistic effect on MCF-7, which is an ER+ and PR+ cell line, but not for the triple-negative MDA-MB-231 cells. These different responses could be due to biological differences between luminal A and triple-negative breast cancer subtypes, which involve variations in drug uptake, mitochondrial dynamics, and oxidative stress levels [63,64]. The synergistic effect was particularly evident for a combination of 10 μM nisin and 1 μM DOX for 48-h treatment with MCF-7 cells. In comparison with normal cell lines, these nisin concentrations were below the cytotoxic range for fibroblast L929 and HaCaT cells as shown in Table 2. Therefore, these conditions were selected for further studies on drug mechanisms.

**Fig 4:**
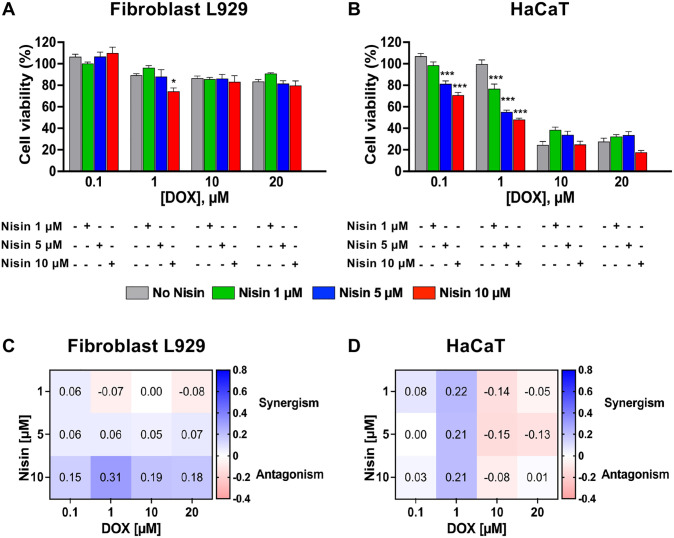
Cell viability (%) of normal cells after treatment with combination of nisin and DOX. **(A)** fibroblast L929 and **(B)** HaCaT cells were treated with nisin, DOX individually or in combination at various concentrations for 24 h. Combination effects in **(C)** fibroblast L929 and **(D)** HaCaT cells were analyzed using Bliss independence drug interaction analysis. Cell viability was determined using MTT assay and data are represented as mean ± SEM (n = 3). *P < 0.05; **P < 0.01; ***P < 0.001 compared with DOX alone.

Furthermore, the combination of nisin and DOX treatment at 24 h showed the viability of normal fibroblast L929 cells that remained above 80% across most combination doses with nisin (1–10 µM) exhibiting no inherent toxicity as shown in Fig 4A, whereas HaCaT cells were more sensitive to the combined treatment, particularly at lower DOX concentration (0.1–1 μM) (Fig 4B). These differences in response may be attributed to variations in cell proliferation rate and intrinsic oxidative stress mechanisms. Although a decrease in cell viability was clearly observed in HaCaT cells with combination treatment, the observed cytotoxicity was primarily associated with DOX, and the synergistic effect observed in both non-cancerous cell lines was less pronounced than that in MCF-7 cells as demonstrated by Bliss independence analysis (Fig 4C and 4D). Thus, the addition of nisin did not significantly potentiate the baseline cytotoxicity of DOX alone. This finding suggests that nisin may serve as a promising chemotherapeutic adjuvant that can enhance the therapeutic efficacy of DOX against breast cancer cells without increasing its off-target toxicity toward normal cells.

### Nisin enhanced cellular uptake of DOX in MCF-7 cells

**Fig 5:**
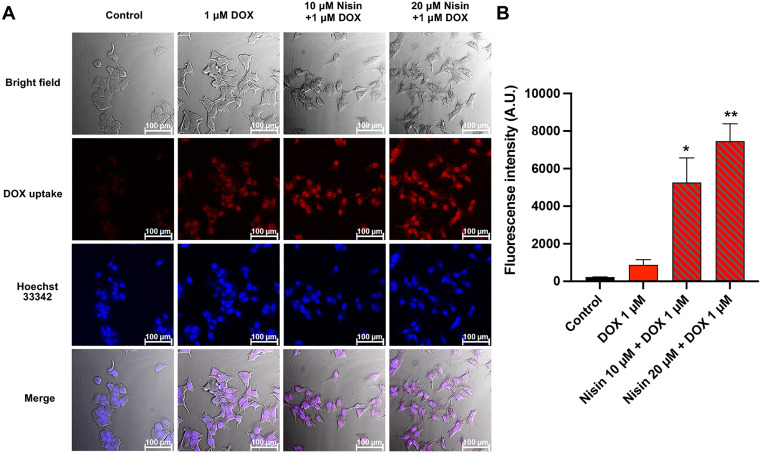
Cellular uptake of DOX by MCF-7 cells. **(A)** Confocal microscopy images of MCF-7 cells incubated with 1 µM DOX alone and combined with nisin at 10 or 20 µM at 37°C for 24 h. Cellular uptake of DOX (red) increased in the presence of nisin. Hoechst 33342 (blue) was used for nuclei staining. **(B)** The fluorescence intensity of DOX (EX/EM 520 nm/600 nm) of MCF-7 after treatment with nisin combined with DOX and free DOX. ^*^P < 0.05; ^**^P < 0.01; compared with DOX alone.

The synergistic effect of nisin and DOX was prominent for MCF-7 cells, when using 10 μM nisin and 1 μM DOX for 48-h treatment. The intracellular trafficking of DOX and nisin combined with DOX was investigated using confocal laser scanning microscopy and spectrophotometry. Due to the membrane permeabilization effect of nisin, the cellular uptake of DOX by MCF-7 cells expectedly enhanced. As shown in Fig 5A, the MCF-7 cells incubated with nisin combined with DOX showed high fluorescence intensity of DOX in the cytoplasm and nuclei, indicating that nisin facilitated DOX uptake inside the cells. By contrast, with treatment of DOX alone, the cellular uptake of DOX was observed with low fluorescence intensity mainly in the cytoplasm. As shown in Fig 5B, the fluorescence intensity was higher in the cells treated with nisin-DOX combination in a dose-dependent manner compared with free DOX. A combined treatment resulted in approximately an 8.5-fold increase in DOX uptake compared with the nisin-free control group at a nisin concentration of 20 µM. Nevertheless, the enhancement of DOX uptake by MDA-MB-231 cells was not clearly demonstrated due to the predominant cytotoxic effect of nisin, which led to cell detachment (see Supplementary Material, S3 Fig).

### Nisin caused cell membrane permeability and enhanced DOX-induced DNA damage

**Fig 6:**
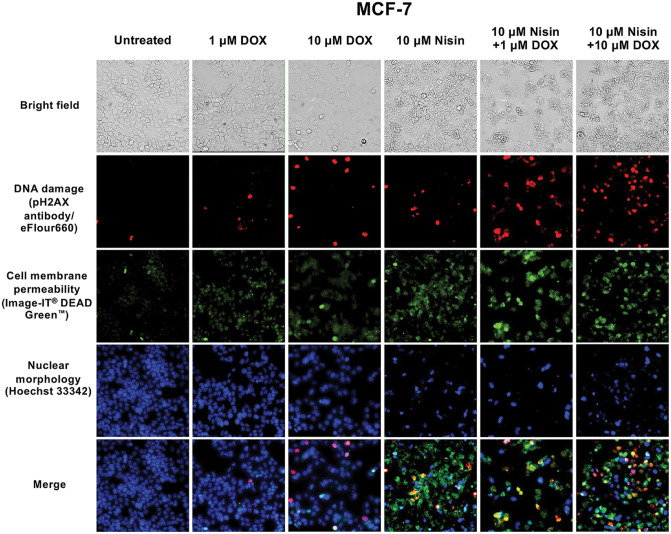
High content imaging of MCF-7 cells in response to DNA damage and cell membrane permeability after treatment with nisin and DOX. MCF-7 cells were treated with indicated concentrations of nisin and DOX alone or in combination for 24 h. Positive results for pH2AX (shown in red), and the Image-iT^®^ DEAD Green^TM^ viability (shown in green) staining indicated DNA damage and compromise in plasma membrane integrity, respectively. Hoechst 33342 stain (shown in blue) was used for nuclear segmentation.

To further examine the effect of nisin on cancer cells, DNA damage of nisin was evaluated by high-content analysis with breast cancer cell lines, MCF-7 and MDA-MB-231. The cells were treated with 10 μM of nisin or 1 μM and 10 μM of DOX for 24 h. DNA double-strand breaks were measured based on detection of fluorescently-labeled antibodies against phosphorylated H2AX (pH2AX) in the cell nucleus, as shown in red. At 10 μM nisin, cells were positive for pH2AX and Image-IT® DEAD Green™viability stain, indicating a compromise in plasma membrane integrity and DNA damage. For 10 μM DOX used as a positive control, both cell lines showed DNA damage and cell membrane permeability as represented by the positive pH2AX and Image-IT® DEAD Green™ viability stain fluorescence (see Fig 6). Our findings revealed that nisin alone at 10 μM did not strongly induce DNA damage in MCF-7 cells but only caused cell membrane permeability. However, co-treatment of nisin and DOX enhanced both DNA damage and cell permeability as indicated by the higher fluorescence intensity of both red and green staining.

**Fig 7:**
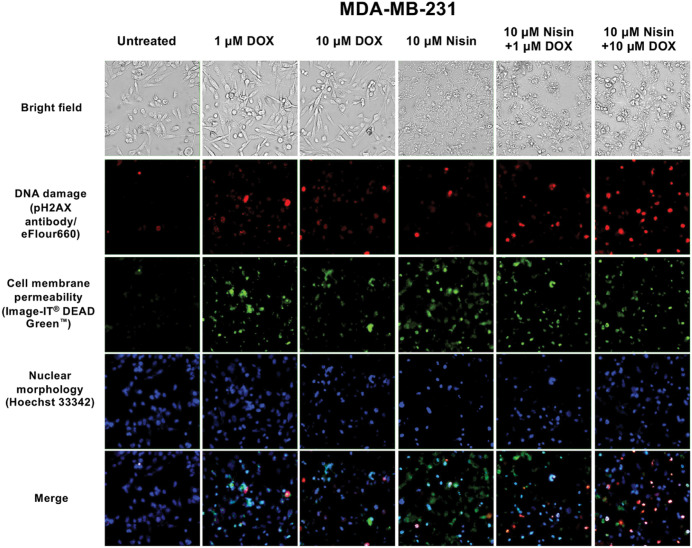
High content imaging of MDA-MB-231 cells in response to DNA damage and cell membrane permeability after treatment with nisin and DOX. MDA-MB-231 cells were treated with indicated concentrations of nisin and DOX alone or in combination for 24 h. Positive results of staining for pH2AX (red), and the Image-iT^®^ DEAD Green^TM^ viability (green) indicated DNA damage and loss of plasma membrane integrity, respectively. Hoechst 33342 stain (shown in blue) was used for nuclear segmentation.

For MDA-MB-231 cells, the phosphorylation of H2AX in the nucleus increased after treatment of DOX (at 1 μM or 10 μM) or nisin at 10 μM alone compared to the control. Similarly to MCF-7, a higher fluorescence intensity in the image was observed in MDA-MB-231 after treatment with the combination of DOX and nisin as shown in Fig 7, indicating DNA damage and cell membrane permeability. It was consistent with previous studies indicating that nisin altered membrane lipid structure and caused pore formation, resulting in depolarization in cell membrane [44]. Altogether, these findings suggested multiple mechanisms of action that nisin enhances cellular sensitivity by disrupting membrane integrity, whereas DOX triggers diverse anticancer pathways−such as genotoxic stress, ROS generation, mitochondrial dysfunction, cell cycle arrest and apoptosis−potentially leading to enhanced efficacy when used in combination [65]. These findings are particularly relevant to hormone receptor-positive breast cancer cells (MCF-7) which typically exhibit moderate sensitivity to DOX due to the presence of efflux transporters and DNA repair mechanisms. Nisin could overcome resistance mechanisms by enhancing DOX accumulation and causing membrane damage, highlighting its potential as a chemotherapeutic adjuvant.

### Combination of nisin and DOX enhanced apoptosis of breast cancer cells

**Fig 8:**
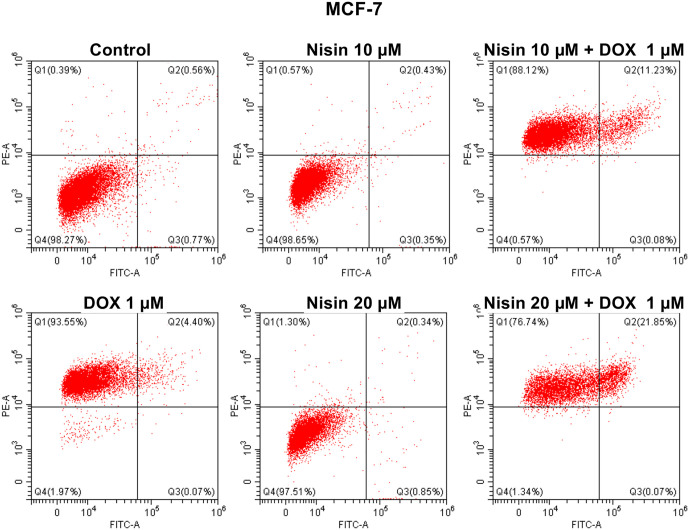
Representative flow cytometry scatter plots of MCF-7 cells after treatment with nisin and DOX. MCF-7 cells were treated with 1 μM DOX and 10 μM or 20 μM nisin for 24 h, followed by Annexin-V and PI staining. A dual-parameter dot plot of Annexin-V (x-axis) and PI (y-axis) were shown. Quadrant definitions: Bottom left—live cells, Top left—necrotic cells, Bottom right—early apoptotic cells, and Top right—late apoptotic cells.

To observe the underlying mechanism of cytotoxic activity induced by nisin and DOX treatment, apoptosis of breast cancer cells was assessed using flow cytometry. Cytotoxic effects of MCF-7 and MDA-MB-231 cells were determined after 24-h treatment with DOX and nisin using Annexin V-FITC/PI staining and flow cytometry. Cell populations including viable, apoptotic and necrotic cells− were quantified using AnnexinV/PI staining. Annexin V + /PI− cells represent early stage of apoptosis, while Annexin V + / PI+ cells indicate the late stages of apoptosis or secondary necrosis. The progression of apoptosis is involved with the exposure of phosphatidylserine (PS) on the outer cell membrane and loss of membrane integrity. For MCF-7 cells, neither apoptosis nor necrosis was clearly observed when treated with nisin alone at 10 and 20 μM, while treatment of 1 μM DOX mainly induced necrosis (~90%) and late apoptosis up to ~4.40%. Notably, the combination of 1 μM DOX and nisin at 10 or 20 μM significantly increased late apoptosis to ~11% and ~22%, respectively (Fig 8), indicating concurrent loss of membrane integrity and apoptosis in MCF-7 cells caused by both therapeutic agents. The enhancement of late apoptosis in MCF-7 after co-treatment of nisin at 10 μM and DOX at 1 μM was correlated with the cytotoxic effect and DNA damage presented in this study.

An increase in late apoptosis observed in MCF-7 cells after co-treatment with nisin and DOX may associate with the membrane-active properties of nisin, which could potentially facilitate intracellular accumulation and enhance cytotoxic effect of co-administered agents. Cationic nisin interacts electrostatically with the negatively charged membranes of cancer cells, resulting in membrane destabilization and increased permeability. This membrane disruption facilitates enhanced intracellular uptake of DOX, which can exert its anticancer activity, primarily through the induction of DNA damage and the activation of multiple apoptotic signaling pathways. These combined effects may contribute to the progression toward late-stage apoptosis, characterized by phosphatidylserine externalization (an early apoptotic marker), together with compromised plasma membrane integrity (a late apoptosis/secondary necrosis marker). Moreover, AMPs, including nisin, have been reported to induce apoptosis by altering of mitochondrial membrane permeability [66]. Disruption of the mitochondrial outer membrane promotes the release of pro-apoptotic factors, leading to caspase cascade activation and amplification of apoptotic death signaling [67].

**Fig 9:**
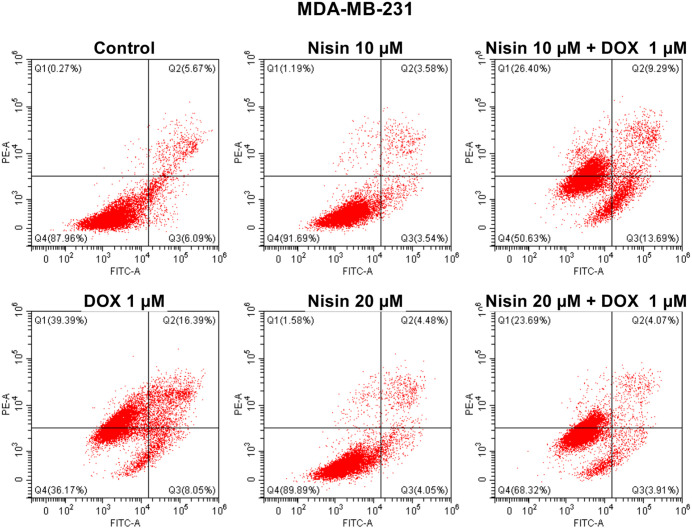
Representative flow cytometry scatter plots of MDA-MB-231 cells after treatment with nisin and DOX. MDA-MB-231 cells were treated with 1 μM DOX and 10 μM or 20 μM nisin for 24 h, followed by Annexin-V and PI staining. A dual-parameter dot plot of Annexin-V (x-axis) and PI (y-axis) were shown. Quadrant definitions: Bottom left—live cells, Top left—necrotic cells, Bottom right—early apoptotic cells, and Top right—late apoptotic cells.

For MDA-MB-231 cells, nisin slightly induced late apoptosis with 3–4%, while DOX at 1 μM triggered mainly necrosis (~30%) and apoptosis (~15%). Unlike MCF-7 cells, co-treatment with nisin and DOX in MDA-MB-231 induced both early and late apoptosis to a comparable extent, without evident dose-dependent response, while the necrotic cells were predominantly observed (see Fig 9), likely due to DOX-induced cytotoxicity. Nevertheless, the apoptotic activity of nisin was previously reported mostly with other cancer cells, such as colorectal cancer [68,69], liver cancer [70] and skin cancer [71], but a few studies have been conducted with breast cancer [54,72].

**Fig 10:**
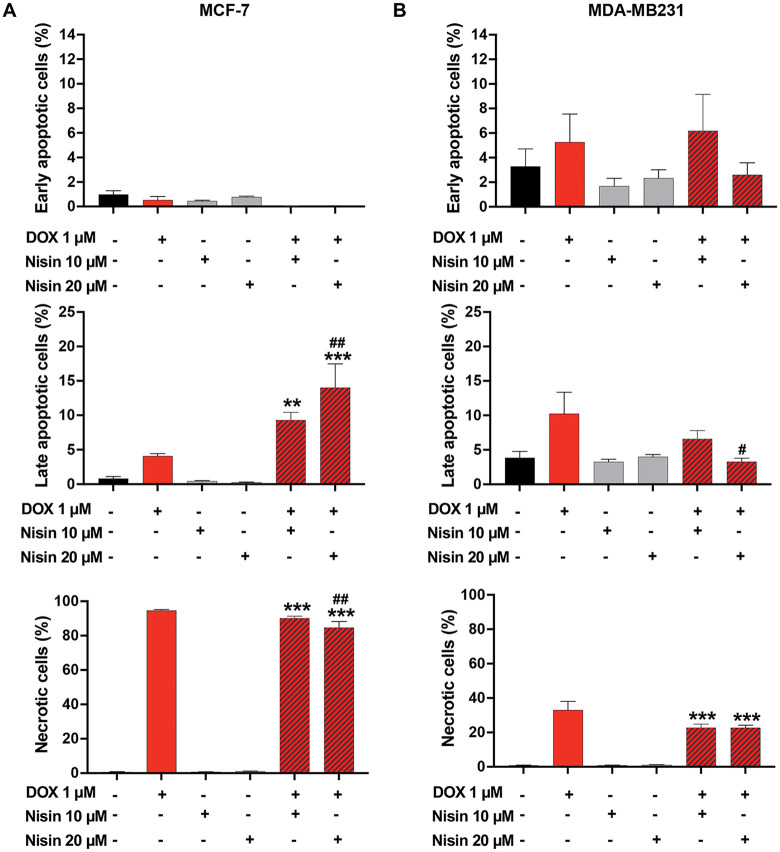
Determination of cell death pathway induced by nisin and DOX in two breast cancer cell lines. The percentages of early apoptotic cells, late apoptotic and necrotic cells in **(A)** MCF-7 and **(B)** MDA-MB-231were determined by flow cytometry. Cells were treated with 1 μM DOX and 10 μM or 20 μM nisin, alone and in combination for 24 h. ^*^P < 0.05; ^**^P < 0.01; ^***^P < 0.001 compared with nisin. ^#^P < 0.05; ^##^P < 0.01; ^###^P < 0.001; compared with DOX alone. Values represent the mean ± SEM (n = 4).

As summarized in Fig 10, MCF-7 and MDA-MB-231 breast cancer cells displayed distinct apoptosis profiles in response to DOX and nisin. The combination of nisin and DOX markedly enhanced apoptosis in MCF-7 cells compared with either treatment alone, whereas the combined effect was comparatively modest in the triple-negative MDA-MB-231 cells. These observations align with previous reports indicating higher sensitivity of MCF-7 cells to DOX-induced ROS production [73].

Although nisin exhibited evident anticancer activity in MDA-MB-231 cells, its combination with DOX did not yield a clearly synergistic response, indicating that the effectiveness of the combined treatment may depend on the molecular features of cancer cells. The significant difference in synergistic potential between MCF-7 and MDA-MB-231 cells, as validated by Tukey’s post-hoc test for multiple comparison tests, highlights the influence of breast cancer molecular subtypes on combination therapy. The synergistic effect was more pronounced in MCF-7 cells, potentially attributable to the membrane-active properties of nisin, whose pore-forming activity has previously been demonstrated in liposomal models. In addition, our cellular findings further support this proposed mechanism. Specifically, high-content analysis using Image-IT® DEAD Green™ staining revealed a compromised plasma membrane integrity in breast cancer cells following nisin treatment (Figs 6 and 7). This loss of membrane integrity correlates with the increase in intracellular DOX accumulation in MCF-7 cells (see Fig 5B), suggesting that nisin-induced permeabilization facilitates drug entry and apoptosis. In contrast, the absence of synergy in MDA-MB-231 cells may reflect the aggressive triple-negative phenotype, associated with its drug-efflux capacity that potentially reduces the membrane-permeabilizing activity of nisin.

This work demonstrated that cationic nisin likely induced membrane permeability in the cancer cell membrane and facilitated DOX uptake, thus enhancing cytotoxic effect of both drugs synergistically. These findings are consistent with previous studies regarding the synergistic effects of nisin when combined with DOX against skin cancer produced by 7,12-dimethylbenz(a)anthracene (DMBA) using a mouse model. The data demonstrated greater improvement in the nisin and DOX combination treatment group, which significantly decreased tumor volume more effectively and enhanced apoptotic cell induction in the tumor tissues of mice [74]. Moreover, previous studies have reported that nisin, when combined with chemotherapeutic agents, such as 5-fluorouracil in skin cancer cells, enhances therapeutic efficacy, exhibiting apoptosis, anti-angiogenesis, and reduced cell proliferation [71]. Although these *in vitro* findings strongly indicate a membrane-based synergistic mechanism, they remain preliminary and indicative of potential therapeutic strategies, requiring further *in vivo* validation to confirm their definitive clinical outcomes.

## Conclusions

This study demonstrated that nisin preferentially interacts with negatively charged phospholipids, which are enriched in cancer cell membranes. Nisin exhibited cytotoxic effects against breast cancer cells, with IC_50_ values ranging from 4.44–7.78 µM and 3.41–5.19 µM, for MCF-7 and MDA-MB-231, while exhibiting substantially lower toxicity toward normal cell lines. At 24–48 h, combined treatment with nisin and DOX showed a synergistic cytotoxic effect particularly in the MCF-7 cells at 10 μM nisin and 1 μM DOX, concentrations that remained below cytotoxic thresholds for normal cells. Our findings suggest that nisin may act as a membrane-active adjuvant that facilitates DOX uptake, thereby enhancing DNA damage and apoptotic pathways. These findings highlight the potential utility of combination approaches to improve anticancer activity while reducing systemic toxicity. Collectively, this work supports the potential of AMPs in combinatorial cancer therapy and identifies nisin as an adjuvant to DOX, particularly in hormone receptor-positive breast cancer. Nevertheless, the present conclusions are limited by the reliance on *in vitro* models, which do not fully reflect the complexity of the tumor microenvironment or intertumoral heterogeneity. Accordingly, further *in vivo* studies, together with pharmacokinetic and pharmacodynamic analyses are essential to confirm the translational feasibility, therapeutic efficacy and safety of nisin-based combination therapy in more complex biological systems.

## Supporting information

S1 Fig Structure of phospholipids used for liposome preparation in dye leakage assay. (TIF)

S2 Fig Cytotoxicity of nisin and DOX against breast cancer and normal cell lines. (TIF)

S3 Fig Cellular uptake of DOX by MDA-MB-231cells demonstrated by Confocal microscopy and fluorescence intensity of DOX. (TIF)
