# Supplementary figures and images for: Synergistic anticancer activity of antimicrobial peptide nisin and doxorubicin against breast cancer cells via modulation of membrane permeability
Source: PLoS One. 2026 Jun 23;21(6):e0352312. doi: 10.1371/journal.pone.0352312 (PMC13289944; doi:10.1371/journal.pone.0352312)

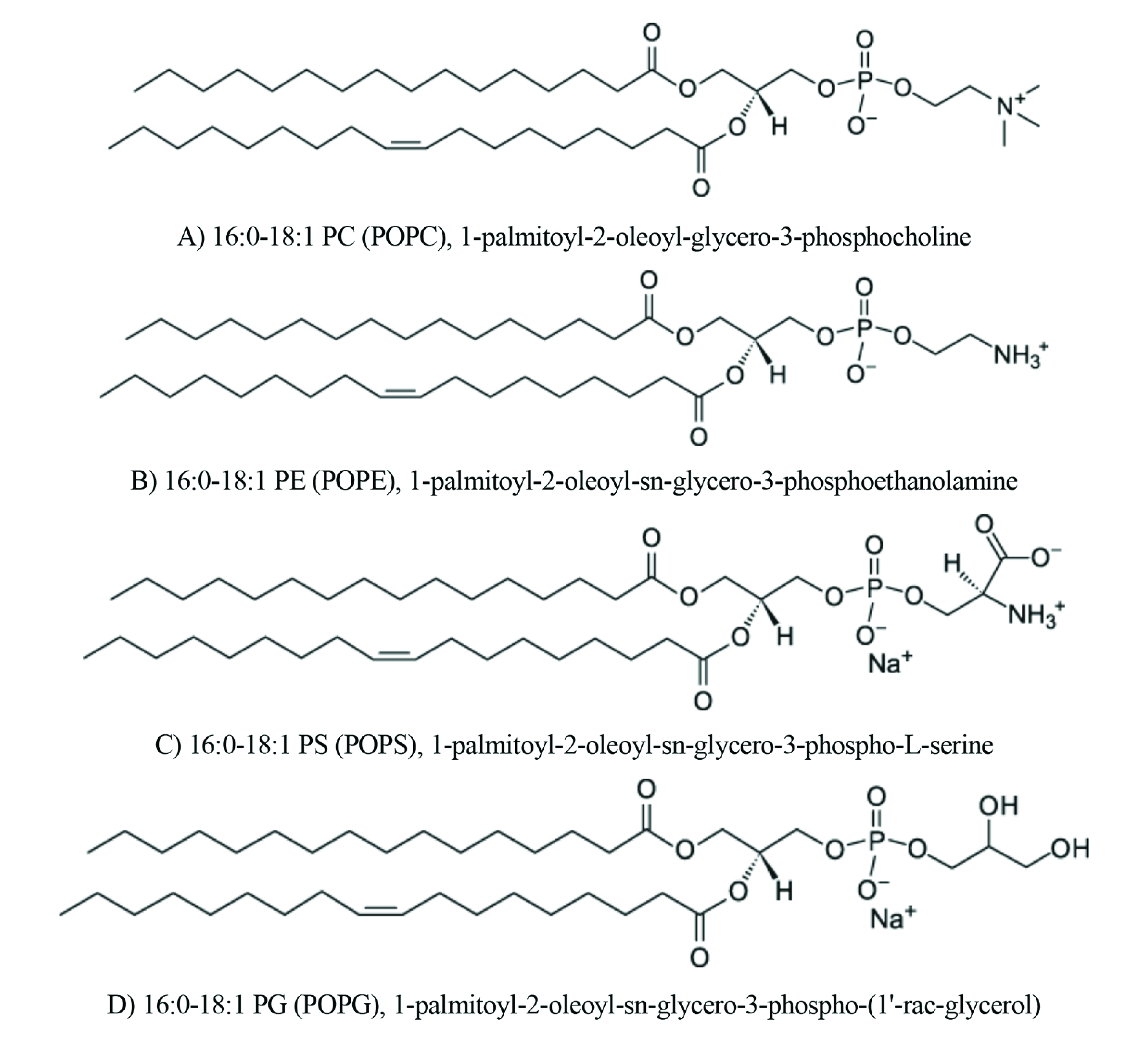

Supplement: S1 Fig — (TIF) [file pone.0352312.s001.tif]

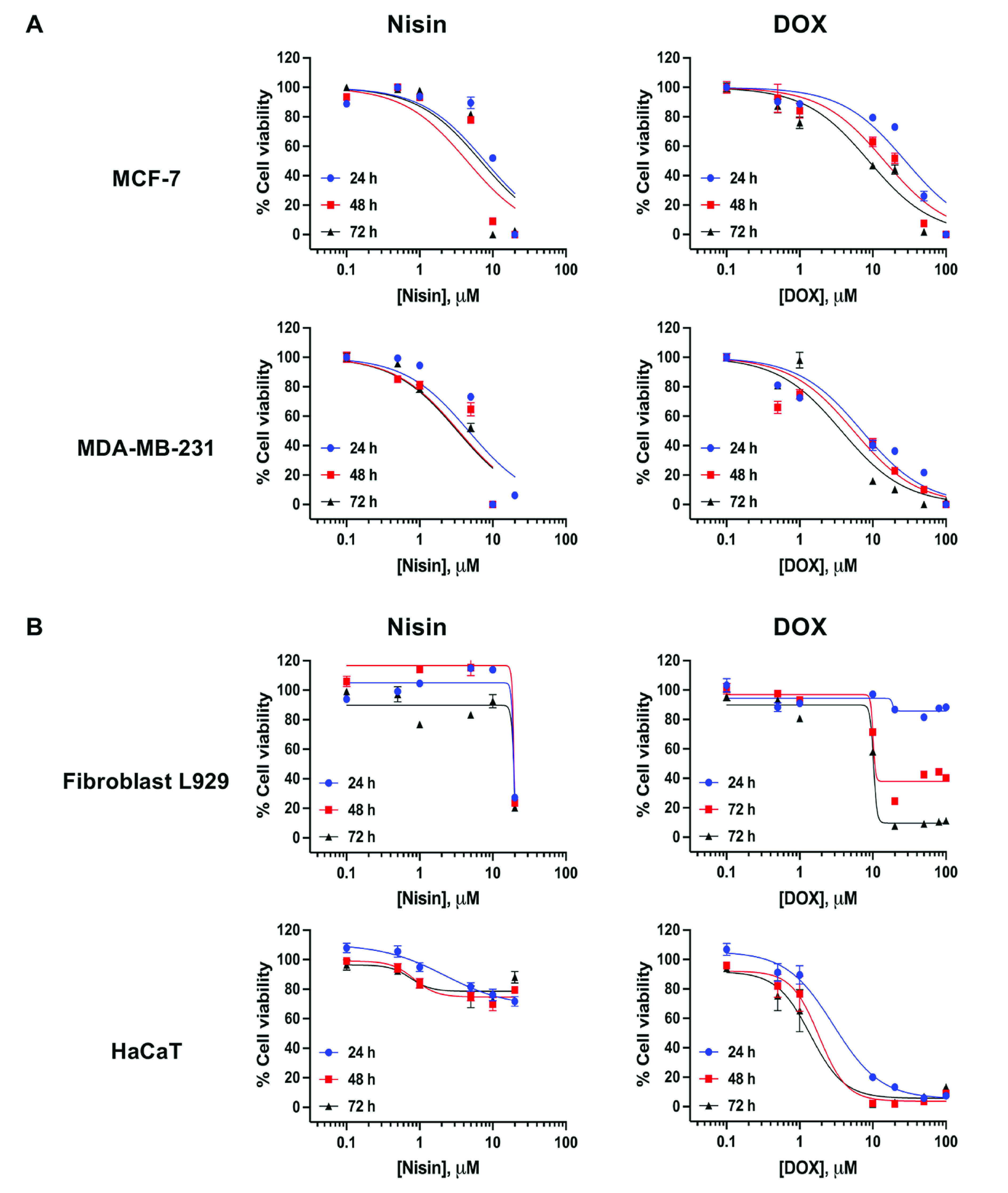

Supplement: S2 Fig — (TIF) [file pone.0352312.s002.tif]

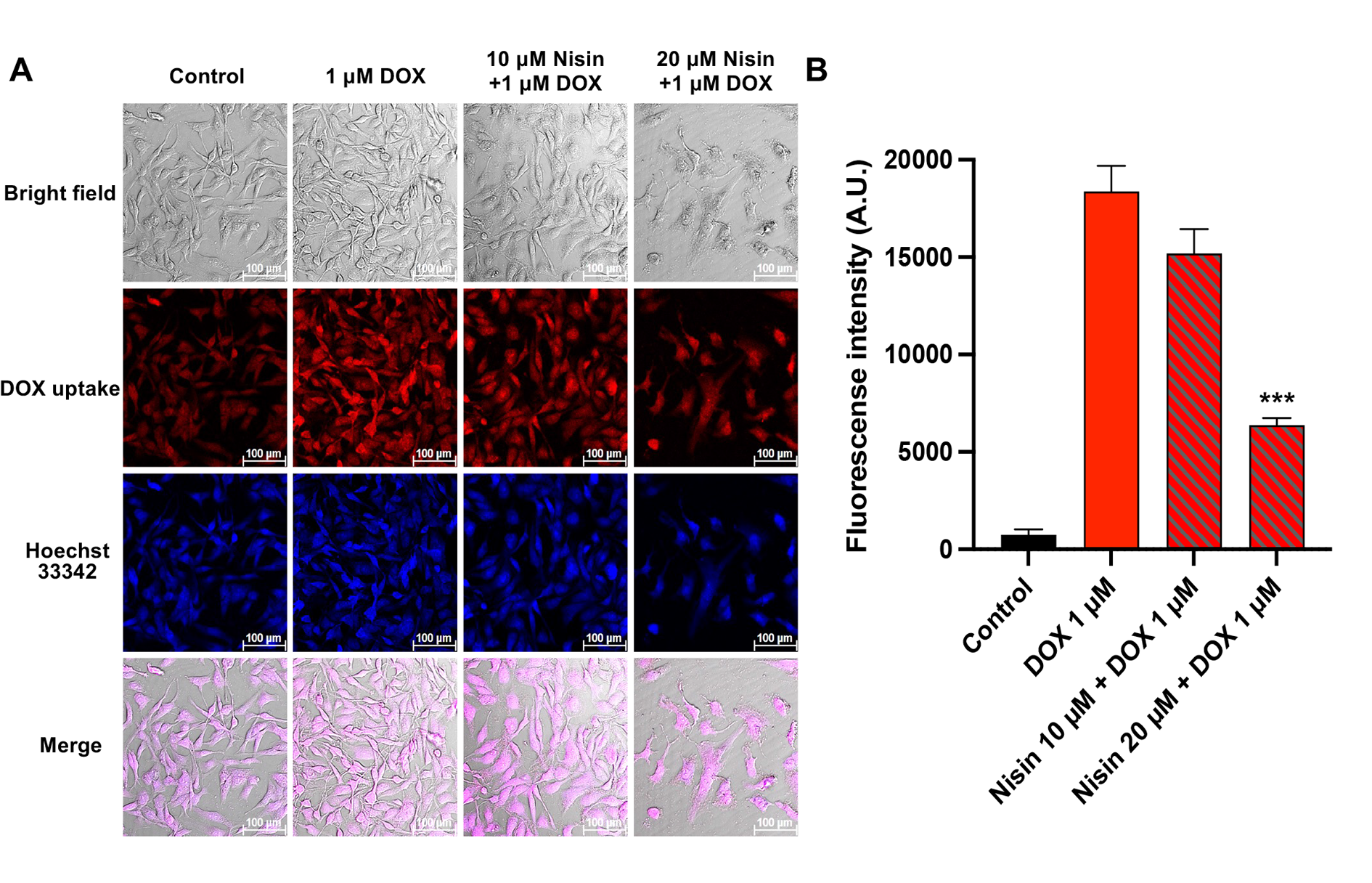

Supplement: S3 Fig — (TIF) [file pone.0352312.s003.tif]
