# Supplementary material for: Synergistic anticancer activity of antimicrobial peptide nisin and doxorubicin against breast cancer cells via modulation of membrane permeability
Source: PLoS One. 2026 Jun 23;21(6):e0352312. doi: 10.1371/journal.pone.0352312 (PMC13289944; doi:10.1371/journal.pone.0352312)
Supplement: Attachment — Submitted filename: Synergistic Anticancer Activity.docx [file pone.0352312.s004.docx]

In this paper entitled “Synergistic Anticancer Activity of Antimicrobial Peptide Nisin and Doxorubicin Against Breast Cancer Cells via Modulation of Membrane Permeability”, Dr. Panchika Prangkio and co-workers demonstrated that nisin preferentially interacts with negatively charged cancer cell membranes, increasing cytotoxicity in breast cancer cells. Combined treatment with nisin and DOX showed a synergistic cytotoxic effect in MCF-7 cells, concentrations that remained below cytotoxic thresholds for normal cells. Nisin increased the permeability of cell membranes, facilitating DOX entry, causing DNA damage and activation of apoptotic pathways in breast cancer cells. Although their conclusions are limited by the reliance on in vitro models, this work supports the potential of AMPs in combinatorial cancer therapy and identifies nisin as an adjuvant to DOX, particularly in hormone receptor-positive breast cancer.
