# Supplementary material for: Synergistic anticancer activity of antimicrobial peptide nisin and doxorubicin against breast cancer cells via modulation of membrane permeability
Source: PLoS One. 2026 Jun 23;21(6):e0352312. doi: 10.1371/journal.pone.0352312 (PMC13289944; doi:10.1371/journal.pone.0352312)
Supplement: Attachment — Submitted filename: Response to reviewers.docx [file pone.0352312.s006.docx]

**Response to reviewers**

Ref.: Ms. No. **PONE-D-26-05856**

Dear Editor,

We appreciate the editor and the reviewers for the insightful comments and suggestion on the manuscript entitled, “**Synergistic Anticancer Activity of Antimicrobial Peptide Nisin and Doxorubicin Against Breast Cancer Cells via Modulation of Membrane Permeability**”. We have revised the manuscript according to the reviewers’ suggestions as marked in blue.
Below we provide the responses to the reviewers’ comments.

**Reviewer #1:** In this paper entitled “Synergistic Anticancer Activity of Antimicrobial Peptide Nisin and Doxorubicin Against Breast Cancer Cells via Modulation of Membrane Permeability”, Dr. Panchika Prangkio and co-workers demonstrated that nisin preferentially interacts with negatively charged cancer cell membranes, increasing cytotoxicity in breast cancer cells. Combined treatment with nisin and DOX showed a synergistic cytotoxic effect in MCF-7 cells, concentrations that remained below cytotoxic thresholds for normal cells. Nisin increased the permeability of cell membranes, facilitating DOX entry, causing DNA damage and activation of apoptotic pathways in breast cancer cells.
Although their conclusions are limited by the reliance on in vitro models, this work supports the potential of AMPs in combinatorial cancer therapy and identifies nisin as an adjuvant to DOX, particularly in hormone receptor-positive breast cancer.
This paper is very interesting, clearly written, the data are nicely presented and supporting their conclusions.

The authors appreciate the reviewer’s comments.

**Reviewer #2:**

- The introduction does not adequately define the existing knowledge gap or highlight the novelty of the study. While the role of antimicrobial peptides and their potential to enhance chemotherapeutic efficacy has already been reported in the literature, the authors need to more clearly articulate the rational and conceptual framework underpinning their work. In particular, the justification for the selected drug combination requires further elaboration. Moreover, the explanation of cancer cell selectivity based solely on differences in membrane charge appears overly simplistic, as it overlooks tumor heterogeneity, which can significantly influence membrane composition and, consequently, drug responsiveness.
- Expanding the introduction to address these points would help more clearly define the research gap and strengthen the overall positioning of the study.

We have revised the introduction section to better define the research gap, particularly the lack of mechanistic evidence regarding nisin-mediated membrane modulation as an adjuvant strategy. (Line 83-91) We have also expanded the rationale for the nisin-DOX combination and addressed the complexity of cancer cell selectivity by discussing tumor heterogeneity and membrane dynamics beyond simple electrostatic interactions. (Line 92-100)

- A major limitation of the study is the narrow experimental model system employed. Although the conclusions presented are reasonable, they should be more cautiously phrased to emphasize that they are speculative and indicative of potential rather than definitive outcomes (e.g., lines 578–580, “in breast cancer”). Furthermore, the use of L929, a non-human fibroblast cell line, as the only control undermines the strength of the results. The authors should consider incorporating human epithelial breast cell models, such as MCF-10A, to provide a more appropriate comparison. To further enhance the translational relevance of the study, the inclusion of patient-derived cells is also strongly recommended.

As suggested by the reviewer, we have revised the manuscript to emphasize that the combined treatment with nisin and DOX showed enhanced cytotoxic effect, particularly in the MCF-7 cells. We have revised the wordings throughout the manuscript to adopt a more cautious and balanced tone, emphasizing potential implications of the findings rather than presenting them as definitive conclusions.

Additionally, we have performed a new set of cytotoxicity experiments with another cell line for appropriate comparison. We investigated the cytotoxicity of nisin and nisin-DOX combination against HaCaT cell line, which is a human epithelial cell model, commonly used as a non-cancerous cell line for evaluating differential responses between normal cells and cancer cells. *(Ref: Devakrishnan P, Nasir NM, Stanslas J, Latif MAM, Ismail AZ, Baharuddin FF. Development and Evaluation of selective nitroxanthone Derivatives: A promising compound for Targeting MCF-7 breast cancer cells. Results Chem. 2025;13: 101998. doi:10.1016/j.rechem.2024.101998)*

Our findings demonstrated consistent safety profiles of nisin in HaCaT keratinocytes, comparable to that observed in L929 cell line as demonstrated in **Table 2.** Some calculated IC50 and Selectivity index values have been updated, based on the comparison with human epithelial cell model, HaCat cell line. However, the cytotoxic effect with combined treatment was stronger in HaCaT than that in L929 fibroblasts as shown in **Fig 4**. Overall, the addition of nisin did not significantly potentiate the baseline cytotoxicity of DOX alone. This finding suggests that nisin may serve as a promising chemotherapeutic adjuvant that can enhance the therapeutic efficacy of DOX against breast cancer cells without increasing its off-target toxicity toward normal cells.

- The main claim of the study is that Nisin enhances Doxorubicin cytotoxicity via increased cancer cell membrane permeabilization. This is not sufficiently supported in the study. As stated in the Methods section, “Determination of membrane permeabilization,” permeabilization was mainly investigated using liposomal model systems and was not directly demonstrated in the breast cancer cell models used in this study. The experiments focus more on drug uptake, apoptosis, and DNA damage, and do not establish membrane permeabilization. The authors should consider either changing their interpretation or providing additional evidence to support their findings.

As we hypothesized that cationic peptide preferentially binds to negatively charged membranes, nisin is likely to exert cytotoxic effect in cancer cells that possess a higher density of negative charges than normal cells. Pore-formation activity of nisin was characterized in liposomal leakage studies, showing the preference of membrane interactions with negatively charged phospholipids.

To provide additional evidence in cellular studies, we used the Image-IT® DEAD Green™ staining (along with DNA damage determination) using high-content analysis to identify cells with compromised plasma membranes as a result of permeabilization of the agents.

We have revised the manuscript to highlight the results from the Image-IT® DEAD Green™ staining and DOX uptake assays, demonstrating the compromised membrane integrity and enhanced permeability in both MCF-7 and MDA-MB-231 cells (Line 591-603).

- The manuscript emphasizes the synergistic effect of Nisin and Doxorubicin. However, it is clearly stated in the manuscript as well as in the presented figures that this effect was significantly more pronounced in MCF-7 cells and was not that consistent in MDA-MB-231 cells. This is a significant variability, and the problem becomes bigger due to the manuscript testing only 2 cell lines. Despite that, the authors conclude and frequently highlight a synergistic therapeutic potential throughout the manuscript. This is an important issue, and the authors should carefully address it. Please revise the manuscript, and state clearly the cell-line-specific nature of their findings to avoid generalizing effects.

We have revised the manuscript to clearly state that the synergistic activity of nisin and DOX is specific for MCF-7 cell line. We have provided a more detailed consideration that nisin may enhance the cytotoxic effect of DOX in MCF-7 cells, potentially through improved cellular uptake of DOX. However, this effect was less evident in MDA-MB-231 cells, as shown in Fig 5 and Supplementary Material, S3 Fig. The synergistic effect was also not observed in either normal cell lines. The authors have more carefully addressed this point in the discussion and conclusion (Line 436-441, 613-616, 619-623).

**Minor comments**
- The upper panel of Figures 5 and 6 (Bright field) should be consistent. Some images are darker.

We have modified the Figures (revised Figs 6-7) according to reviewer’s suggestions.

- The authors should clarify that all experiments were performed with mycoplasma-free cells.

We have provided more details in material and method section (Line 143-145).

- The authors state that in the MTT protocol, cells were seeded and allowed to attach for 24 h. Afterwards they were incubated with Nisin or DOX, in DMEM medium for 24h. The authors should specify whether this medium was the aforementioned recipe (subsection “Cell culture”) or whether it was the starvation medium. If so, the authors should state whether this was plain DMEM medium or if it contained anything else. Additionally, it is common practice in MTT assays to incubate the cells in the dark after adding MTT. This was not stated in the manuscript. If this was done, please add this information. If not, the authors should provide a brief explanation as to why.

We have provided more details in material and method section (Line 179-190).

- It is not clearly specified whether post-hoc tests were performed for multiple comparisons. The statistical analysis section would strongly benefit from additional details.

We have now updated the statistical analysis section in the materials and methods to specify that Tukey’s post-hoc test was performed following one-way ANOVA to determine significant differences between all experimental groups. We have also ensured that all p-values in the figures reflect these post-hoc comparisons (Line 196-198) and addressed in Discussion (Line 591-593).

- Figure S3 has some contrasting results. This should be stated in the main text. Additionally, whether or not statistical analyses were performed should be stated. And also shown in the figure.

We have moved the Supplementary Figure of cytotoxicity test in normal cell to main text and performed the statistical analyses as suggested. We also included the new data of additional normal epithelial HaCaT cell line. (See Fig. 4 in the revised manuscript).
