# Supplementary material for: Synergistic anticancer activity of antimicrobial peptide nisin and doxorubicin against breast cancer cells via modulation of membrane permeability
Source: PLoS One. 2026 Jun 23;21(6):e0352312. doi: 10.1371/journal.pone.0352312 (PMC13289944; doi:10.1371/journal.pone.0352312)
Supplement: Peer review history

# Peer review: Decision Letter 0

**DOI:** 10.1371/journal.pone.0352312.r001

**Authors:** Alexandra Lianou

23 Mar 2026

PONE-D-26-05856

Synergistic Anticancer Activity of Antimicrobial Peptide Nisin and Doxorubicin Against Breast Cancer Cells via Modulation of Membrane Permeability

PLOS One

Dear Dr. Prangkio,

Thank you for submitting your manuscript to PLOS ONE. After careful consideration, we feel that it has merit but does not fully meet PLOS ONE’s publication criteria as it currently stands. Therefore, we invite you to submit a revised version of the manuscript that addresses the points raised during the review process.

==============================

ACADEMIC EDITOR:

The authors are kindly requested to address the comments raised by the reviewers, either by proceeding with the suggested revisions or by providing the rationale for not doing so.

Specifically, one of the reviewers (Reviewer 2) has raised some serious concerns regarding the technical soundness of the manuscript, pointing out certain limitations of the conducted research, mainly with reference to (i) the experimental model system used (i.e. non-inclusion of human epithelial breast cell models) and (ii) the generalized interpretation of the results pertinent to the synergistic effects of the bacteriocin nisin and doxorubicin (which may be cell line-specific, a parameter that is not sufficiently acknowledged by the authors).

==============================

Please submit your revised manuscript by May 07 2026 11:59PM. If you will need more time than this to complete your revisions, please reply to this message or contact the journal office at plosone@plos.org. When you're ready to submit your revision, log on to https://www.editorialmanager.com/pone/ and select the 'Submissions Needing Revision' folder to locate your manuscript file.

Please include the following items when submitting your revised manuscript:

- A letter that responds to each point raised by the academic editor and reviewer(s). You should upload this letter as a separate file labeled 'Response to Reviewers'.
- A marked-up copy of your manuscript that highlights changes made to the original version. You should upload this as a separate file labeled 'Revised Manuscript with Track Changes'.
- An unmarked version of your revised paper without tracked changes. You should upload this as a separate file labeled 'Manuscript'.

If you would like to make changes to your financial disclosure, please include your updated statement in your cover letter. Guidelines for resubmitting your figure files are available below the reviewer comments at the end of this letter.

If applicable, we recommend that you deposit your laboratory protocols in protocols.io to enhance the reproducibility of your results. Protocols.io assigns your protocol its own identifier (DOI) so that it can be cited independently in the future. For instructions see: https://journals.plos.org/plosone/s/submission-guidelines#loc-laboratory-protocols. Additionally, PLOS ONE offers an option for publishing peer-reviewed Lab Protocol articles, which describe protocols hosted on protocols.io. Read more information on sharing protocols at https://plos.org/protocols?utm_medium=editorial-email&utm_source=authorletters&utm_campaign=protocols.

We look forward to receiving your revised manuscript.

Kind regards,

Alexandra Lianou, M.Sc., Ph.D.

Academic Editor

PLOS One

Journal Requirements:

When submitting your revision, we need you to address these additional requirements.

1. Please ensure that your manuscript meets PLOS ONE's style requirements, including those for file naming. The PLOS ONE style templates can be found at

https://journals.plos.org/plosone/s/file?id=wjVg/PLOSOne_formatting_sample_main_body.pdf and

https://journals.plos.org/plosone/s/file?id=ba62/PLOSOne_formatting_sample_title_authors_affiliations.pdf

2. Thank you for stating the following in the Acknowledgments Section of your manuscript:

" The project was supported by Materials Science Research Center, Faculty of Science, Chiang Mai University and Thailand Science Research and Innovation (TSRI): Fundamental Fund 2024 (Grant No. FF043/2567). Financial supports were provided by the Science Achievement Scholarship of Thailand (SAST) (to C.P.), the Development and Promotion of Science and Technology Talents Project (DPST) Scholarship (to S.N.) and Chiang Mai University Presidential Scholarship (to U.A.). The authors would like to acknowledge the Department of Chemistry, Faculty of Science, and Medical Science Research Equi"

We note that you have provided additional information within the Acknowledgements Section that is not currently declared in your Funding Statement. Please note that funding information should not appear in the Acknowledgments section or other areas of your manuscript. We will only publish funding information present in the Funding Statement section of the online submission form.

Please remove any funding-related text from the manuscript and let us know how you would like to update your Funding Statement. Currently, your Funding Statement reads as follows:

"The project was partially supported by Materials Science Research Center, Faculty of Science, Chiang Mai University and Thailand Science Research and Innovation (TSRI): Fundamental Fund 2024 (Grant No. FF043/2567). Financial supports were provided by the Science Achievement Scholarship of Thailand (SAST) (to C.P.), the Development and Promotion of Science and Technology Talents Project (DPST) Scholarship (to S.N.) and Chiang Mai University Presidential Scholarship (to U.A.)."

Please include your amended statements within your cover letter; we will change the online submission form on your behalf.

3. Thank you for stating the following financial disclosure:

"The project was partially supported by Materials Science Research Center, Faculty of Science, Chiang Mai University and Thailand Science Research and Innovation (TSRI): Fundamental Fund 2024 (Grant No. FF043/2567). Financial supports were provided by the Science Achievement Scholarship of Thailand (SAST) (to C.P.), the Development and Promotion of Science and Technology Talents Project (DPST) Scholarship (to S.N.) and Chiang Mai University Presidential Scholarship (to U.A.). "

Please state what role the funders took in the study.  If the funders had no role, please state: "The funders had no role in study design, data collection and analysis, decision to publish, or preparation of the manuscript."

If this statement is not correct you must amend it as needed.

Please include this amended Role of Funder statement in your cover letter; we will change the online submission form on your behalf.

4. Please include captions for your Supporting Information files at the end of your manuscript, and update any in-text citations to match accordingly. Please see our Supporting Information guidelines for more information: http://journals.plos.org/plosone/s/supporting-information.

5. If the reviewer comments include a recommendation to cite specific previously published works, please review and evaluate these publications to determine whether they are relevant and should be cited. There is no requirement to cite these works unless the editor has indicated otherwise.

[Note: HTML markup is below. Please do not edit.]

Reviewers' comments:

Reviewer's Responses to Questions

**Comments to the Author**

1. Is the manuscript technically sound, and do the data support the conclusions?

The manuscript must describe a technically sound piece of scientific research with data that supports the conclusions. Experiments must have been conducted rigorously, with appropriate controls, replication, and sample sizes. The conclusions must be drawn appropriately based on the data presented.

Reviewer #1: Yes

Reviewer #2: Partly

**********

2. Has the statistical analysis been performed appropriately and rigorously?

Reviewer #1: Yes

Reviewer #2: Yes

**********

3. Have the authors made all data underlying the findings in their manuscript fully available?

The PLOS Data policy requires authors to make all data underlying the findings described in their manuscript fully available without restriction, with rare exception (please refer to the Data Availability Statement in the manuscript PDF file). The data should be provided as part of the manuscript or its supporting information, or deposited to a public repository. For example, in addition to summary statistics, the data points behind means, medians and variance measures should be available. If there are restrictions on publicly sharing data—e.g. participant privacy or use of data from a third party—those must be specified.

Reviewer #1: Yes

Reviewer #2: No

**********

4. Is the manuscript presented in an intelligible fashion and written in standard English?

PLOS ONE does not copyedit accepted manuscripts, so the language in submitted articles must be clear, correct, and unambiguous. Any typographical or grammatical errors should be corrected at revision, so please note any specific errors here.

Reviewer #1: Yes

Reviewer #2: Yes

**********

5. Review Comments to the Author

Please use the space provided to explain your answers to the questions above. You may also include additional comments for the author, including concerns about dual publication, research ethics, or publication ethics. (Please upload your review as an attachment if it exceeds 20,000 characters)

Reviewer #1: In this paper entitled “Synergistic Anticancer Activity of Antimicrobial Peptide Nisin and Doxorubicin Against Breast Cancer Cells via Modulation of Membrane Permeability”, Dr. Panchika Prangkio and co-workers demonstrated that nisin preferentially interacts with negatively charged cancer cell membranes, increasing cytotoxicity in breast cancer cells. Combined treatment with nisin and DOX showed a synergistic cytotoxic effect in MCF-7 cells, concentrations that remained below cytotoxic thresholds for normal cells. Nisin increased the permeability of cell membranes, facilitating DOX entry, causing DNA damage and activation of apoptotic pathways in breast cancer cells. Although their conclusions are limited by the reliance on in vitro models, this work supports the potential of AMPs in combinatorial cancer therapy and identifies nisin as an adjuvant to DOX, particularly in hormone receptor-positive breast cancer.

This paper is very interesting, clearly written, the data are nicely presented and supporting their conclusions.

Reviewer #2: - The introduction does not adequately define the existing knowledge gap or highlight the novelty of the study. While the role of antimicrobial peptides and their potential to enhance chemotherapeutic efficacy has already been reported in the literature, the authors need to more clearly articulate the rationale and conceptual framework underpinning their work. In particular, the justification for the selected drug combination requires further elaboration. Moreover, the explanation of cancer cell selectivity based solely on differences in membrane charge appears overly simplistic, as it overlooks tumor heterogeneity, which can significantly influence membrane composition and, consequently, drug responsiveness. Expanding the introduction to address these points would help more clearly define the research gap and strengthen the overall positioning of the study.

- A major limitation of the study is the narrow experimental model system employed. Although the conclusions presented are reasonable, they should be more cautiously phrased to emphasize that they are speculative and indicative of potential rather than definitive outcomes (e.g., lines 578–580, “in breast cancer”). Furthermore, the use of L929, a non-human fibroblast cell line, as the only control undermines the strength of the results. The authors should consider incorporating human epithelial breast cell models, such as MCF-10A, to provide a more appropriate comparison. To further enhance the translational relevance of the study, the inclusion of patient-derived cells is also strongly recommended.

- The main claim of the study is that Nisin enhances Doxorubicin cytotoxicity via increased cancer cell membrane permeabilization. This is not sufficiently supported in the study. As stated in the Methods section, “Determination of membrane permeabilization,” permeabilization was mainly investigated using liposomal model systems and was not directly demonstrated in the breast cancer cell models used in this study. The experiments focus more on drug uptake, apoptosis, and DNA damage, and do not establish membrane permeabilization. The authors should consider either changing their interpretation or providing additional evidence to support their findings.

- The manuscript emphasizes the synergistic effect of Nisin and Doxorubicin. However, it is clearly stated in the manuscript as well as in the presented figures that this effect was significantly more pronounced in MCF-7 cells, and was not that consistent in MDA-MB-231 cells. This is a significant variability, and the problem becomes bigger due to the manuscript testing only 2 cell lines. Despite that, the authors conclude and frequently highlight a synergistic therapeutic potential throughout the manuscript. This is an important issue, and the authors should carefully address it. Please revise the manuscript, and state clearly the cell-line-specific nature of their findings to avoid generalizing effects.

Minor comments

- The upper panel of Figures 5 and 6 (Bright field) should be consistent. Some images are darker.

- The authors should clarify that all experiments were performed with mycoplasma-free cells.

- The authors state that in the MTT protocol, cells were seeded and allowed to attach for 24 h. Afterwards they were incubated with Nisin or DOX, in DMEM medium for 24h. The authors should specify whether this medium was the aforementioned recipe (subsection “Cell culture”) or whether it was the starvation medium. If so, the authors should state whether this was plain DMEM medium or if it contained anything else. Additionally, it is common practice in MTT assays to incubate the cells in the dark after adding MTT. This was not stated in the manuscript. If this was done, please add this information. If not, the authors should provide a brief explanation as to why.

- It is not clearly specified whether post-hoc tests were performed for multiple comparisons. The statistical analysis section would strongly benefit from additional details.

- Figure S3 has some contrasting results. This should be stated in the main text. Additionally, whether or not statistical analyses were performed should be stated. And also shown in the figure.

-

**********

6. PLOS authors have the option to publish the peer review history of their article (what does this mean?). If published, this will include your full peer review and any attached files.

If you choose “no”, your identity will remain anonymous but your review may still be made public.

**Do you want your identity to be public for this peer review?**  For information about this choice, including consent withdrawal, please see our Privacy Policy.

Reviewer #1: No

Reviewer #2: No

**********

[NOTE: If reviewer comments were submitted as an attachment file, they will be attached to this email and accessible via the submission site. Please log into your account, locate the manuscript record, and check for the action link "View Attachments". If this link does not appear, there are no attachment files.]

To ensure your figures meet our technical requirements, please review our figure guidelines: https://journals.plos.org/plosone/s/figures

You may also use PLOS’s free figure tool, NAAS, to help you prepare publication quality figures: https://journals.plos.org/plosone/s/figures#loc-tools-for-figure-preparation.

NAAS will assess whether your figures meet our technical requirements by comparing each figure against our figure specifications.

Attachment Submitted filename: Synergistic Anticancer Activity.docx

# Author comment: Author response to Decision Letter 1

**DOI:** 10.1371/journal.pone.0352312.r002

12 May 2026

Response to reviewers

Ref.: Ms. No. PONE-D-26-05856

Dear Editor,

We appreciate the editor and the reviewers for the insightful comments and suggestion on the manuscript entitled, “Synergistic Anticancer Activity of Antimicrobial Peptide Nisin and Doxorubicin Against Breast Cancer Cells via Modulation of Membrane Permeability”. We have revised the manuscript according to the reviewers’ suggestions as marked in blue.

Below we provide the responses to the reviewers’ comments.

Reviewer #1: In this paper entitled “Synergistic Anticancer Activity of Antimicrobial Peptide Nisin and Doxorubicin Against Breast Cancer Cells via Modulation of Membrane Permeability”, Dr. Panchika Prangkio and co-workers demonstrated that nisin preferentially interacts with negatively charged cancer cell membranes, increasing cytotoxicity in breast cancer cells. Combined treatment with nisin and DOX showed a synergistic cytotoxic effect in MCF-7 cells, concentrations that remained below cytotoxic thresholds for normal cells. Nisin increased the permeability of cell membranes, facilitating DOX entry, causing DNA damage and activation of apoptotic pathways in breast cancer cells.

Although their conclusions are limited by the reliance on in vitro models, this work supports the potential of AMPs in combinatorial cancer therapy and identifies nisin as an adjuvant to DOX, particularly in hormone receptor-positive breast cancer.

This paper is very interesting, clearly written, the data are nicely presented and supporting their conclusions.

The authors appreciate the reviewer’s comments.

Reviewer #2:

- The introduction does not adequately define the existing knowledge gap or highlight the novelty of the study. While the role of antimicrobial peptides and their potential to enhance chemotherapeutic efficacy has already been reported in the literature, the authors need to more clearly articulate the rational and conceptual framework underpinning their work. In particular, the justification for the selected drug combination requires further elaboration. Moreover, the explanation of cancer cell selectivity based solely on differences in membrane charge appears overly simplistic, as it overlooks tumor heterogeneity, which can significantly influence membrane composition and, consequently, drug responsiveness.

- Expanding the introduction to address these points would help more clearly define the research gap and strengthen the overall positioning of the study.

We have revised the introduction section to better define the research gap, particularly the lack of mechanistic evidence regarding nisin-mediated membrane modulation as an adjuvant strategy. (Line 83-91) We have also expanded the rationale for the nisin-DOX combination and addressed the complexity of cancer cell selectivity by discussing tumor heterogeneity and membrane dynamics beyond simple electrostatic interactions. (Line 92-100)

- A major limitation of the study is the narrow experimental model system employed. Although the conclusions presented are reasonable, they should be more cautiously phrased to emphasize that they are speculative and indicative of potential rather than definitive outcomes (e.g., lines 578–580, “in breast cancer”). Furthermore, the use of L929, a non-human fibroblast cell line, as the only control undermines the strength of the results. The authors should consider incorporating human epithelial breast cell models, such as MCF-10A, to provide a more appropriate comparison. To further enhance the translational relevance of the study, the inclusion of patient-derived cells is also strongly recommended.

As suggested by the reviewer, we have revised the manuscript to emphasize that the combined treatment with nisin and DOX showed enhanced cytotoxic effect, particularly in the MCF-7 cells. We have revised the wordings throughout the manuscript to adopt a more cautious and balanced tone, emphasizing potential implications of the findings rather than presenting them as definitive conclusions.

Additionally, we have performed a new set of cytotoxicity experiments with another cell line for appropriate comparison. We investigated the cytotoxicity of nisin and nisin-DOX combination against HaCaT cell line, which is a human epithelial cell model, commonly used as a non-cancerous cell line for evaluating differential responses between normal cells and cancer cells. (Ref: Devakrishnan P, Nasir NM, Stanslas J, Latif MAM, Ismail AZ, Baharuddin FF. Development and Evaluation of selective nitroxanthone Derivatives: A promising compound for Targeting MCF-7 breast cancer cells. Results Chem. 2025;13: 101998. doi:10.1016/j.rechem.2024.101998)

Our findings demonstrated consistent safety profiles of nisin in HaCaT keratinocytes, comparable to that observed in L929 cell line as demonstrated in Table 2. Some calculated IC50 and Selectivity index values have been updated, based on the comparison with human epithelial cell model, HaCat cell line. However, the cytotoxic effect with combined treatment was stronger in HaCaT than that in L929 fibroblasts as shown in Fig 4. Overall, the addition of nisin did not significantly potentiate the baseline cytotoxicity of DOX alone. This finding suggests that nisin may serve as a promising chemotherapeutic adjuvant that can enhance the therapeutic efficacy of DOX against breast cancer cells without increasing its off-target toxicity toward normal cells.

- The main claim of the study is that Nisin enhances Doxorubicin cytotoxicity via increased cancer cell membrane permeabilization. This is not sufficiently supported in the study. As stated in the Methods section, “Determination of membrane permeabilization,” permeabilization was mainly investigated using liposomal model systems and was not directly demonstrated in the breast cancer cell models used in this study. The experiments focus more on drug uptake, apoptosis, and DNA damage, and do not establish membrane permeabilization. The authors should consider either changing their interpretation or providing additional evidence to support their findings.

As we hypothesized that cationic peptide preferentially binds to negatively charged membranes, nisin is likely to exert cytotoxic effect in cancer cells that possess a higher density of negative charges than normal cells. Pore-formation activity of nisin was characterized in liposomal leakage studies, showing the preference of membrane interactions with negatively charged phospholipids.

To provide additional evidence in cellular studies, we used the Image-IT® DEAD Green™ staining (along with DNA damage determination) using high-content analysis to identify cells with compromised plasma membranes as a result of permeabilization of the agents.

We have revised the manuscript to highlight the results from the Image-IT® DEAD Green™ staining and DOX uptake assays, demonstrating the compromised membrane integrity and enhanced permeability in both MCF-7 and MDA-MB-231 cells (Line 591-603).

- The manuscript emphasizes the synergistic effect of Nisin and Doxorubicin. However, it is clearly stated in the manuscript as well as in the presented figures that this effect was significantly more pronounced in MCF-7 cells and was not that consistent in MDA-MB-231 cells. This is a significant variability, and the problem becomes bigger due to the manuscript testing only 2 cell lines. Despite that, the authors conclude and frequently highlight a synergistic therapeutic potential throughout the manuscript. This is an important issue, and the authors should carefully address it. Please revise the manuscript, and state clearly the cell-line-specific nature of their findings to avoid generalizing effects.

We have revised the manuscript to clearly state that the synergistic activity of nisin and DOX is specific for MCF-7 cell line. We have provided a more detailed consideration that nisin may enhance the cytotoxic effect of DOX in MCF-7 cells, potentially through improved cellular uptake of DOX. However, this effect was less evident in MDA-MB-231 cells, as shown in Fig 5 and Supplementary Material, S3 Fig. The synergistic effect was also not observed in either normal cell lines. The authors have more carefully addressed this point in the discussion and conclusion (Line 436-441, 613-616, 619-623).

Minor comments

- The upper panel of Figures 5 and 6 (Bright field) should be consistent. Some images are darker.

We have modified the Figures (revised Figs 6-7) according to reviewer’s suggestions.

- The authors should clarify that all experiments were performed with mycoplasma-free cells.

We have provided more details in material and method section (Line 143-145).

- The authors state that in the MTT protocol, cells were seeded and allowed to attach for 24 h. Afterwards they were incubated with Nisin or DOX, in DMEM medium for 24h. The authors should specify whether this medium was the aforementioned recipe (subsection “Cell culture”) or whether it was the starvation medium. If so, the authors should state whether this was plain DMEM medium or if it contained anything else. Additionally, it is common practice in MTT assays to incubate the cells in the dark after adding MTT. This was not stated in the manuscript. If this was done, please add this information. If not, the authors should provide a brief explanation as to why.

We have provided more details in material and method section (Line 179-190).

- It is not clearly specified whether post-hoc tests were performed for multiple comparisons. The statistical analysis section would strongly benefit from additional details.

We have now updated the statistical analysis section in the materials and methods to specify that Tukey’s post-hoc test was performed following one-way ANOVA to determine significant differences between all experimental groups. We have also ensured that all p-values in the figures reflect these post-hoc comparisons (Line 196-198) and addressed in Discussion (Line 591-593).

- Figure S3 has some contrasting results. This should be stated in the main text. Additionally, whether or not statistical analyses were performed should be stated. And also shown in the figure.

We have moved the Supplementary Figure of cytotoxicity test in normal cell to main text and performed the statistical analyses as suggested. We also included the new data of additional normal epithelial HaCaT cell line. (See Fig. 4 in the revised manuscript).

Attachment Submitted filename: Response to reviewers.docx

# Peer review: Decision Letter 1

**DOI:** 10.1371/journal.pone.0352312.r003

**Authors:** Alexandra Lianou, Alexandra Lianou

9 Jun 2026

Synergistic Anticancer Activity of Antimicrobial Peptide Nisin and Doxorubicin Against Breast Cancer Cells via Modulation of Membrane Permeability

PONE-D-26-05856R1

Dear Dr. Prangkio,

We’re pleased to inform you that your manuscript has been judged scientifically suitable for publication and will be formally accepted for publication once it meets all outstanding technical requirements.

Within one week, you’ll receive an e-mail detailing the required amendments. When these have been addressed, you’ll receive a formal acceptance letter and your manuscript will be scheduled for publication.

An invoice will be generated when your article is formally accepted. Please note, if your institution has a publishing partnership with PLOS and your article meets the relevant criteria, all or part of your publication costs will be covered. Please make sure your user information is up-to-date by logging into Editorial Manager at Editorial Manager® and clicking the ‘Update My Information' link at the top of the page. For questions related to billing, please contact billing support.

If your institution or institutions have a press office, please notify them about your upcoming paper to help maximize its impact. If they’ll be preparing press materials, please inform our press team as soon as possible -- no later than 48 hours after receiving the formal acceptance. Your manuscript will remain under strict press embargo until 2 pm Eastern Time on the date of publication. For more information, please contact onepress@plos.org.

Kind regards,

Alexandra Lianou, M.Sc., Ph.D.

Academic Editor

PLOS One

Additional Editor Comments (optional):

According to the reviewers' and the Academic editor's evaluation, the revised manuscript has been significantly improved and all previously raised comments have been adequately addressed. Hence, the revised version of the manuscript is acceptable for publication.

Reviewers' comments:

Reviewer's Responses to Questions

**Comments to the Author**

1. If the authors have adequately addressed your comments raised in a previous round of review and you feel that this manuscript is now acceptable for publication, you may indicate that here to bypass the “Comments to the Author” section, enter your conflict of interest statement in the “Confidential to Editor” section, and submit your "Accept" recommendation.

Reviewer #1: All comments have been addressed

Reviewer #2: All comments have been addressed

**********

2. Is the manuscript technically sound, and do the data support the conclusions?

The manuscript must describe a technically sound piece of scientific research with data that supports the conclusions. Experiments must have been conducted rigorously, with appropriate controls, replication, and sample sizes. The conclusions must be drawn appropriately based on the data presented.

Reviewer #1: Yes

Reviewer #2: Yes

**********

3. Has the statistical analysis been performed appropriately and rigorously?

Reviewer #1: Yes

Reviewer #2: Yes

**********

4. Have the authors made all data underlying the findings in their manuscript fully available?

The PLOS Data policy requires authors to make all data underlying the findings described in their manuscript fully available without restriction, with rare exception (please refer to the Data Availability Statement in the manuscript PDF file). The data should be provided as part of the manuscript or its supporting information, or deposited to a public repository. For example, in addition to summary statistics, the data points behind means, medians and variance measures should be available. If there are restrictions on publicly sharing data—e.g. participant privacy or use of data from a third party—those must be specified.

Reviewer #1: Yes

Reviewer #2: Yes

**********

5. Is the manuscript presented in an intelligible fashion and written in standard English?

PLOS ONE does not copyedit accepted manuscripts, so the language in submitted articles must be clear, correct, and unambiguous. Any typographical or grammatical errors should be corrected at revision, so please note any specific errors here.

Reviewer #1: Yes

Reviewer #2: Yes

**********

6. Review Comments to the Author

Please use the space provided to explain your answers to the questions above. You may also include additional comments for the author, including concerns about dual publication, research ethics, or publication ethics. (Please upload your review as an attachment if it exceeds 20,000 characters)

Reviewer #1: The paper is interesting, clearly written, the data are nicely presented and support their conclusions.

Reviewer #2: The authors have sufficiently addressed my comments. The introduction has been substantially strengthened, and also statistical analyses have been clarified, with the manuscript now more appropriately emphasizing the cell-line-specific nature of the observed synergistic effects. The additional cellular data and revised interpretation are sufficient to support the authors' conclusions at the current level. I have no further major concerns and consider the manuscript acceptable for publication.

**********

7. PLOS authors have the option to publish the peer review history of their article (what does this mean?). If published, this will include your full peer review and any attached files.

If you choose “no”, your identity will remain anonymous but your review may still be made public.

**Do you want your identity to be public for this peer review?**  For information about this choice, including consent withdrawal, please see our Privacy Policy.

Reviewer #1: No

Reviewer #2: No

**********

# Editor report: Acceptance letter

**DOI:** 10.1371/journal.pone.0352312.r004

**Authors:** Alexandra Lianou, Alexandra Lianou

PONE-D-26-05856R1

PLOS One

Dear Dr. Prangkio,

I'm pleased to inform you that your manuscript has been deemed suitable for publication in PLOS One. Congratulations! Your manuscript is now being handed over to our production team.

At this stage, our production department will prepare your paper for publication. This includes ensuring the following:

* All references, tables, and figures are properly cited

* All relevant supporting information is included in the manuscript submission,

* There are no issues that prevent the paper from being properly typeset

You will receive further instructions from the production team, including instructions on how to review your proof when it is ready. Please keep in mind that we are working through a large volume of accepted articles, so please give us a few days to review your paper and let you know the next and final steps.

Lastly, if your institution or institutions have a press office, please let them know about your upcoming paper now to help maximize its impact. If they'll be preparing press materials, please inform our press team within the next 48 hours. Your manuscript will remain under strict press embargo until 2 pm Eastern Time on the date of publication. For more information, please contact onepress@plos.org.

You will receive an invoice from PLOS for your publication fee after your manuscript has reached the completed accept phase. If you receive an email requesting payment before acceptance or for any other service, this may be a phishing scheme. Learn how to identify phishing emails and protect your accounts at https://explore.plos.org/phishing.

If we can help with anything else, please email us at customercare@plos.org.

Thank you for submitting your work to PLOS ONE and supporting open access.

Kind regards,

PLOS ONE Editorial Office Staff

on behalf of

Dr. Alexandra Lianou

Academic Editor

PLOS One
